# Analysis of the copy number profiles of several tumor samples from the same patient reveals the successive steps in tumorigenesis

**DOI:** 10.1186/gb-2010-11-7-r76

**Published:** 2010-07-22

**Authors:** Eric Letouzé, Yves Allory, Marc A Bollet, François Radvanyi, Frédéric Guyon

**Affiliations:** 1 INSERM, UMR-S 973, MTi, Université Paris Diderot - Paris 7, 35 rue Hélène Brion, 75205 Paris Cedex 13, France; 2 Institut Curie, Centre de Recherche, Paris, F-75248 France; 3 CNRS, UMR 144, 26 rue d'Ulm, 75248 Paris Cedex 05, France; 4 INSERM, Unité 955, Créteil F-94000, France; 5 AP-HP, Groupe Hospitalier Albert Chenevier - Henri Mondor, Département de Pathologie, Créteil F-94000, France; 6 Département d'Oncologie Radiothérapie, Institut Curie, 26 rue d'Ulm, 75248 Paris Cedex 05, France

## Abstract

We present a computational method, TuMult, for reconstructing the sequence of copy number changes driving carcinogenesis, based on the analysis of several tumor samples from the same patient. We demonstrate the reliability of the method with simulated data, and describe applications to three different cancers, showing that TuMult is a valuable tool for the establishment of clonal relationships between tumor samples and the identification of chromosome aberrations occurring at crucial steps in cancer progression.

## Background

It is now widely accepted that cancers arise from an accumulation of genetic and epigenetic alterations, through which cells acquire the properties required for malignancy [1]. These alterations - mutations, chromosomal aberrations and aberrant DNA methylation - are inherently random and undirected, consistent with a model of clonal evolution [2], in which advanced tumors result from the clonal expansion of a single cell of origin and the sequential selection of sublines with additional alterations conferring a growth advantage. As a result, the tumor finally detected in clinical conditions usually displays a complex pattern of genetic alterations. As we generally only have data for a single time point in cancer progression (the time of surgery), the standard approach to elucidating the various steps in tumorigenesis has been to compare genetic alterations in tumors from different patients, with cancers of different histological stages and grades. Early alterations are defined as changes observed at all stages, whereas late events are alterations associated exclusively with advanced stages. The first model of the accumulation of genetic events was proposed by Fearon and Vogelstein, who described a five-step model for the development of colorectal cancer [3,4]. With the advent of pangenomic copy number analyses, computational methods were developed for inferring models of cancer progression through the analysis of copy number changes in a set of tumors from various patients [5-10]. However, attempts to find simple models for other types of cancer were hindered by the high diversity of genetic alterations encountered, even in tumors considered to be clinically and pathologically homogeneous, due to the existence of several carcinogenesis pathways and the absence of validation on real examples of tumor progression in a single patient.

A more straightforward approach to unraveling the succession of steps in cancer development whilst taking into account the diverse situations in which a healthy cell may become cancerous is to analyze several samples from a single patient at different locations or different time points during the disease. In this way, it is possible to reconstruct the sequence of alterations really occurring in a patient, rather than a theoretical model generated by the comparison of heterogeneous samples [11]. Such analyses are possible only if several biopsy specimens are available for the same patient, either because a premalignant condition led to prospective biopsies [11], or because recurrences or metastases have been removed following excision of the primary tumor. Bladder cancer is a particularly useful model system for this kind of study because of its high recurrence rate (50 to 60% of patients with non muscle-invasive bladder tumors develop one or more recurrences after transurethral resection). Analyses of copy number alterations in several metachronous or synchronous multifocal urothelial tumors have been carried out with microsatellite markers [12,13] or by comparative genomic hybridization (CGH) [14-17]. Based on chromosomal aberrations common to several samples, the authors of these studies were able to reconstruct the relationships between samples, and showed these tumors to have a monoclonal origin.

Such analyses may be carried out manually when only a few events are involved. However, automated approaches are required to ensure that the maximum benefit is gained from the most recent technologies for high-definition pangenomic copy number analysis (> 10^6 ^probes on the most recent generation of arrays). We describe here the first computational method, TuMult, for reconstructing the lineage of the tumors, together with the sequence of chromosomal events occurring during tumorigenesis, based on the high-resolution mapping of common breakpoints in the copy number profiles of several samples from the same patient. We demonstrate the reliability of the method, through the analysis of simulated tumor progression data. We then apply TuMult to three experimental data sets (BAC array CGH and SNP data), corresponding to bladder tumor recurrences, pairs of primary breast carcinomas and ipsilateral recurrences [18], and metastatic samples from different anatomic sites within individual prostate cancer patients [19].

## Results

### Reconstructing the tumor progression tree from the identification of common chromosome breakpoints

**Figure 1:**
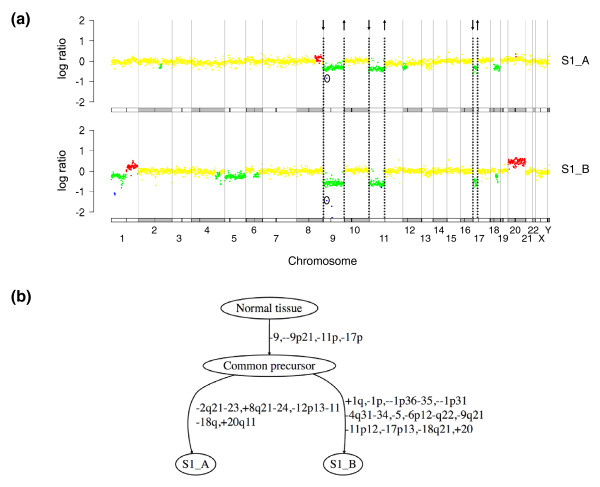
**Principle of tumor progression tree reconstruction**. **(a) **CGH log ratio profiles of two bladder tumors from the same patient, with color code as follows: homozygous deletions in blue, losses in green, normal regions in yellow, and gains in red. Chromosomes are delineated by gray vertical lines and a schematic representation of chromosomes and centromeres is drawn below each profile. Chromosome breakpoints common to both samples are indicated by dashed lines, with an arrow representing the sign of each breakpoint. For greater clarity, the common breakpoints on either side of the one-BAC homozygous deletion at 9p21 are not drawn. This common aberration is instead circled in each profile. **(b) **Tumor progression tree reconstructed for the two samples. Common breakpoints define early aberrations occurring in the common precursor of the two samples. Chromosome aberrations specific to each tumor are placed on subsequent edges.

Two tumors descended from the same initial cancerous cell generally have a number of genetic alterations in common, these changes having occurred before the separation of the two clones. They also display specific genetic alterations that occurred independently in each clone after their separation. A comparison of the alterations in each clone can thus be used to reconstruct the sequence of chromosomal events giving rise to each tumor (Figure 1). Logically, clones separating later in the tumorigenesis process should have more genetic events in common than those separating earlier in this process. This is the simple reasoning underlying our methodology. The TuMult algorithm reconstructs the tumor lineage tree from the leaves (tumors) to the root (normal cell), by iterative grouping of the two closest nodes in terms of chromosome breakpoints. Simultaneously, the copy number profile of each intermediate node, corresponding to an ancestral tumor clone, is reconstructed at each step of the algorithm (see Materials and methods for details).

**Figure 2:**
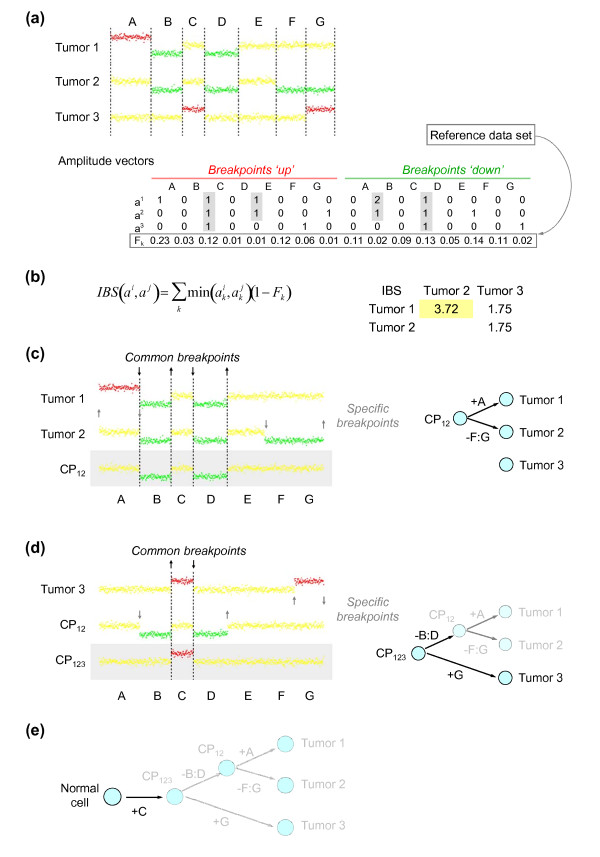
**Overview of the TuMult algorithm**. **(a) **Discretized copy number profiles of three tumors from the same patient (yellow, 'normal copy number'; green, 'loss'; red, 'gain'). The eight breakpoints identified in the samples (dashed lines) divide the chromosome into seven 'homogeneous segments', A to G, in which copy number is constant in any sample. The profiles can be represented as amplitude vectors (see Materials and methods), in which 'up' and 'down' breakpoints are distinguished by their position in the vector. A common breakpoint (gray shading) appears as a non-zero value at the same position in the amplitude matrix. The frequency F_k _of each breakpoint is calculated from a reference data set of independent samples. **(b) **An identical breakpoint score (*IBS*), characterizing the similarity of two profiles in terms of chromosome breakpoints, is calculated for each pair of samples, and the pair displaying the highest level of similarity is selected. **(c) **The copy number profile of the common precursor of the two samples is reconstructed based on their common breakpoints, represented by dashed lines and black arrows. Edges are added between the common precursor (CP) and the two nodes, labeled with the aberrations defined by their specific breakpoints, represented by gray arrows. Note that a breakpoint may be both common and specific, if its amplitude is larger in one of the samples, like the 'down' breakpoint between segments A and B in this example. **(d) **Steps (b) and (c) are iterated until there is only one node left in the front. **(e) **A 'normal cell' node has been added above the common ancestor of all tumors.

As chromosomal aberrations accumulate during tumor progression, several aberrations may affect the same region of the chromosome in succession. An aberration common to two samples will therefore be missed if it is partly affected by a subsequent aberration overlapping the same region. However, common breakpoints remain recognizable in most cases (as illustrated in Figure 2d), making it possible to infer the initial genetic alteration occurring in the common precursor of the samples. Indeed, a breakpoint is only erased if a breakpoint of the opposite sign occurs at the same location, and such events are likely to be rare. We therefore decided to use chromosome breakpoints, rather than chromosome aberrations, for reconstruction of the tumor progression trees.

The input data for TuMult are the discretized copy number profiles of several tumors from the same patient. Before reconstructing the tumor progression tree, all the chromosome breakpoints identified in all the samples from the patient are used to delineate 'homogeneous segments' (see Materials and methods), and the copy number profile of each sample is represented as a breakpoint amplitude vector (Figure 2a), representing the absolute values of shifts in copy number between segments. 'Up' (increase in copy number) and 'down' (decrease in copy number) breakpoints are differentiated in terms of their position in the amplitude vector. A common breakpoint, defined as a breakpoint of the same sign and at the same genomic location in two samples, is thus easy to spot as a non-zero value at the same position in the amplitude vectors for these two samples.

At each step in the algorithm, the two nodes that separated most recently in the tumor lineage, and which therefore have the largest number of chromosome events in common, are joined. We have introduced an identical breakpoint score (IBS) for quantifying the similarity of two profiles on the basis of their amplitude vectors. This score is obtained by adding the amplitudes of the breakpoints common to both profiles, weighted down by the frequency of each breakpoint in a reference data set. Very frequent breakpoints are more likely to occur independently by chance in the two samples, and are therefore less informative than rarer breakpoints. This score is used at each step in the inference of the tree to identify the two closest nodes (Figure 2b). The copy number profile of the common precursor of the two nodes is then inferred from the breakpoints they have in common (see Materials and methods), and the events specific to each tumor, deduced from the breakpoints observed in only one of the two tumors, are associated with the edges between each tumor and the common precursor (Figure 2c). This process is iterated until there is only one node left: the common precursor of all the samples (Figure 2d). A node corresponding to the normal cell is eventually added at the top of the tree, together with an edge from the normal cell to the common precursor of all samples (Figure 2e).

### Evaluation of the performance of the algorithm with simulated data

**Figure 3:**
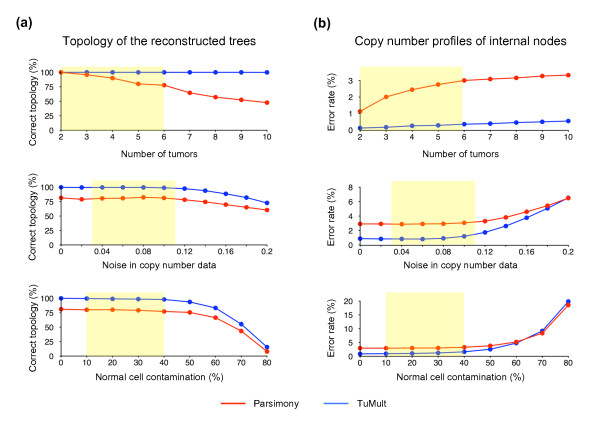
**Evaluation of the performance of TuMult and the parsimony method with simulated data**. Simulated data were generated to evaluate the performance of TuMult and the parsimony method for the reconstruction of tumor progression trees. The performance of each algorithm was assessed under each set of conditions by generating 1,000 random trees and calculating **(a) **the percentage of the reconstructed trees with the correct topology, and **(b)**, for the trees with the correct topology, the percentage of probes with incorrect copy number status in the internal nodes. Simulations were carried out for different numbers of tumors (upper panel), various levels of noise in the data (middle panel), and various proportions of normal cells in the samples (bottom panel). The number of tumors analyzed (between 2 and 6), together with the levels of noise (between 0.03 and 0.11) and contamination (10 to 40%) estimated for our experimental data are represented by yellow areas.

The performance of the TuMult algorithm was evaluated by generating simulated tumor progression trees for various numbers of tumors, with different levels of noise and normal cell contamination (see Materials and methods). The trees were simulated by repeating three steps: 1, picking up a node from the leaves of the tree under construction; 2, adding two edges to this node, with a random number of aberrations at random genomic locations on each edge; 3, calculating the resulting profiles for the two descending nodes. This process produces a tree of random topology, with random copy number profiles for all the nodes. For each condition, 1,000 random trees were generated, and the copy number profiles of the leaves were used as an input for the TuMult algorithm. The ability of Tumult to reconstruct the correct tree topology was investigated by calculating, in each set of conditions, the percentage of the reconstructed trees with a topology identical to that of the original simulated tree (Figure 3a). For trees with the correct topology, the ability of TuMult to reconstruct the correct copy number profiles for ancestral nodes was evaluated by calculating the proportion of probes with an incorrect copy number status in these nodes (Figure 3b). The performance of TuMult was benchmarked by analyzing the same simulated data by the parsimony method [20]. This method was originally designed for phylogeny reconstruction. It reconstructs the tree with the minimum number of changes, each species being characterized by a set of discrete characters. We adapted this method to the reconstruction of tumor progression trees by considering each segment in the copy number profile as a character, with a discrete number of values (-2 to 2).

As the number of tumors increases, so does the number of successive steps in the simulated trees and, hence, the probability of successive aberrations overlapping the same region, or the same set of probes being altered by independent events on different edges. As a result, the performance of the parsimony method rapidly decreases as a function of the number of tumors (Figure 3, upper panel). By contrast, the TuMult algorithm inferred the correct topology in all simulations, whatever the number of tumors involved, with a very small increase in error rate for the copy number profiles of the internal nodes.

The impact of noise and normal cell contamination were evaluated on simulated trees with five leaves. The results of TuMult and the parsimony method were unaffected by noise with a standard deviation below 0.10, and normal cell contamination levels below 40%. The performance of both algorithms then declined. The decline was slightly faster for the TuMult algorithm, which performed a little less well than the parsimony method in terms of error rate at very high noise levels (> 0.2; Figure 3b, middle panel) or at high levels of contamination (> 60%; Figure 3b, bottom panel).

However, in the range of noise and contamination expected for data of reasonably good quality, such as the data analyzed below (noise < 0.11 and contamination < 40%), the TuMult algorithm was much more efficient than the parsimony method, giving the correct topology in > 98% of cases, with an error rate in the internal node profiles of < 1.6%.

### Application to the study of bladder carcinogenesis

Five patients for whom two to four bladder cancer samples were available were analyzed with the TuMult algorithm. In four cases, we had metachronous samples obtained at different times during the course of the disease. In the remaining case (P3), we had samples from different synchronous tumors removed from a cystectomy specimen (Table 1). The tumors from three patients (P1 to P3) were analyzed with BAC arrays (2,385 probes), and the tumors from two patients (P4 and P5) were analyzed with Illumina SNP arrays (373,397 probes).

**Table 1:** Clinical data for the 13 bladder samples analyzed with the TuMult algorithm

Sample	**Patient**	Sex	Stage	Grade	Surgery time	Copy number analysis
S1_A	P1	M	T2	G2	t0	BAC array-CGH
S1_B	P1	M	T1	G3	t0 + 21.8 months	BAC array-CGH
S2_A	P2	M	T3	G3	t0	BAC array-CGH
S2_B	P2	M	T3	G3	t0 + 2.1 months	BAC array-CGH
S3_A	P3	M	T4	G3	t0 + 157 months	BAC array-CGH
S3_B	P3	M	T4	G3	t0 + 157 months	BAC array-CGH
S3_C	P3	M	T4	G3	t0 + 157 months	BAC array-CGH
S3_D	P3	M	T4	G3	t0 + 157 months	BAC array-CGH
S4_A	P4	F	T1	G2	t0	SNP array
S4_B	P4	F	T1	G3	t0 + 14.4 months	SNP array
S5_A	P5	M	Ta	G1	t0	SNP array
S5_B	P5	M	Ta	G1	t0 + 7.8 months	SNP array
S5_C	P5	M	T3	G3	t0 + 10.3 months	SNP array

In the 'Surgery time' column, t0 refers to the time of occurrence of the primary tumor.

**Figure 4:**
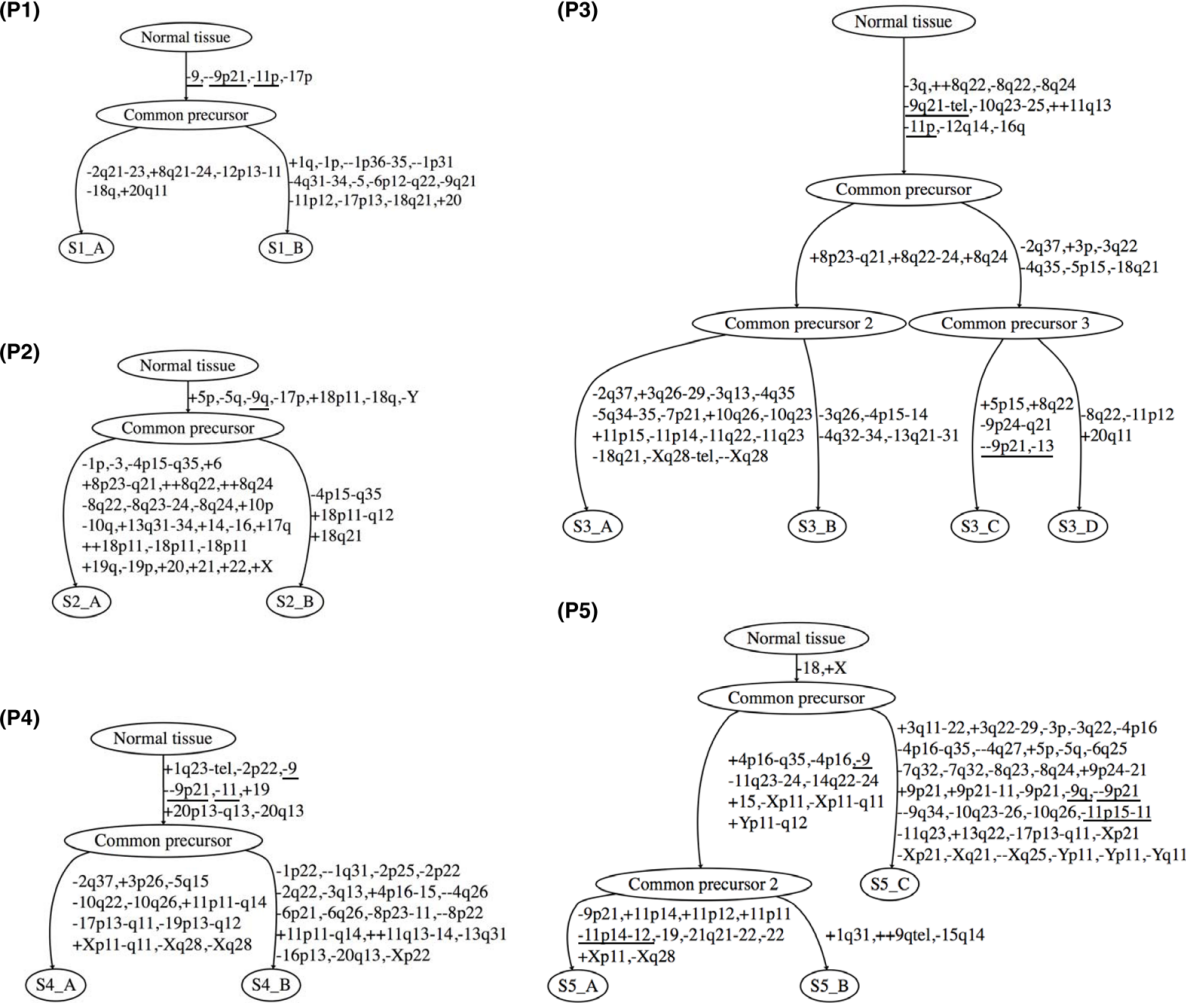
**Bladder tumor progression trees reconstructed with the TuMult algorithm**. Thirteen samples from five patients were analyzed with the TuMult algorithm to reconstruct the tumor lineage and sequence of chromosomal aberrations in each case. Aberrations are annotated as follows: (--) homozygous deletions, (-) losses, (+) gains, (++) amplicons. Aberration boundaries are indicated in terms of chromosome cytobands. Tumor progression trees with aberrations indicated in terms of homogeneous segments are available, together with the segment description tables, from the TuMult web page [43]. Losses of chromosome arms 9q and 11p are underlined, along with homozygous deletions of 9p21.3. The aberrations -9q and -11p were the most frequent early events in the tumor progression trees. In addition, -9q and -11p occurred together on the same edge significantly more frequently than would be expected by chance (*P *= 0.0025). This was also true of -11p and -9p21.3 (*P *= 0.012). Clinical details for each sample can be found in Table 1.

The hypothesis of a monoclonal origin for all samples was supported by the large number of shared chromosome aberrations (Figure 4) in all patients except P5. In P5, we found that only a small proportion of the aberrations present in tumor S5_C were common to S5_A and S5_B, raising questions about whether tumor S5_C resulted from the same initial clone as S5_A and S5_B but diverged early, or whether it had a different origin but acquired a few similar events by chance.

Interestingly, sample S5_C was obtained from an invasive tumor, whereas the other two samples from P5 were from superficial Ta tumors. Although S5_C was detected less than 3 months after S5_B, our analysis shows that these two samples displayed only a weak clonal relationship, if any. Note that our findings regarding clonality are highly consistent with clonality determinations based on the partial identity score proposed by Bollet *et al. *[18] (Additional file 1).

No linear evolution, in which one tumor could be identified as the direct descendant of another tumor, was observed. Instead, each tumor displayed a subset of specific events occurring after the divergence of the tumors. In some cases, the primary tumor may have many more aberrations than the recurrence, as found for S2_A and S2_B or S5_A and S5_B, consistent with the finding of van Tilborg *et al. *[13] that tumor complexity is not correlated with the chronological order in which tumors are clinically detected. Thus, the aberrations displayed by the primary tumor do not reliably reflect the initial steps of tumor progression. By contrast, tumor progression trees make it possible to identify the events occurring at the start of tumorigenesis, even from a set of very complex samples, as in patient P3, in which a subset of ten early aberrations was identified, including two amplicons reported to be frequent in bladder cancer [21-25], at 11q13.3 (*Cyclin D1*) and 8q22.2 (no known oncogene). The number of cancers studied was too small for inference, with a satisfactory level of statistical confidence, of the chronology of chromosomal events in bladder cancer, but the most frequently observed events on the initial edge of the tumor progression trees were -9q (in four out of five tumor progression trees), which is known to be one of the earliest steps in most bladder cancers [26-28], and -11p (in three out of five tumor progression trees). Finally, as the aberrations observed on the same edge of a tumor progression tree presumably occurred during the same time period, we investigated the co-occurrence of the most frequent aberrations in bladder cancer on the 21 edges of our five tumor progression trees (see Materials and methods). Despite the limited statistical power of our test, due to the small number of trees, -11p was shown to occur on the same edge as -9q (*P *= 0.0025) and --9p21.3 (*CDKN2A *tumor suppressor; *P *= 0.012) significantly more frequently than would be expected by chance. This suggests a possible synergic effect of these three aberrations on tumor growth. Alternatively, the co-occurrence of such events may have a mechanistic cause, such as frequent chromosome rearrangement, as between chromosomes 1 and 16 in Ewing sarcoma [29].

### Application to the study of breast carcinogenesis

**Figure 5:**
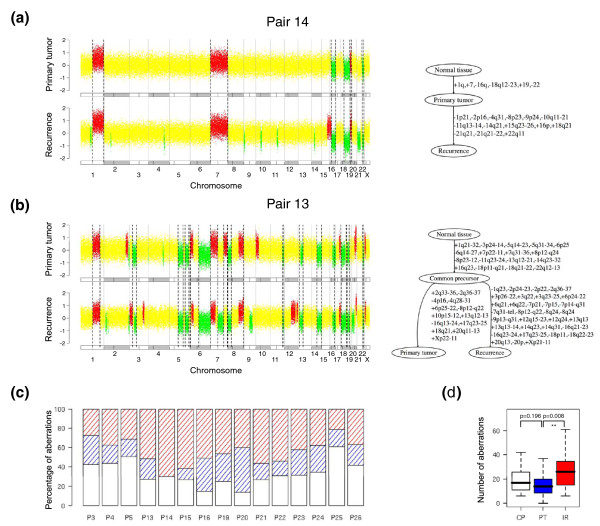
**Accumulation of chromosome aberrations during breast cancer progression**. Fifteen pairs of primary breast carcinomas and ipsilateral recurrences were analyzed with the TuMult algorithm. Patients are denoted as in the original article by Bollet *et al. *[18]. **(a) **In patient P14, all the aberrations of the primary tumor were found in the recurrence, consistent with a linear evolution. **(b) **In patient P13, both the primary tumor and the recurrence display specific events, implying that the recurrence was not directly descended from the primary tumor. **(c) **The proportion of all the aberrations in the tree occurring before the common precursor (white), between the common precursor and the primary tumor (blue hatched) or between the common precursor and the recurrence (red hatched) is presented for each of the 15 patients. P14 is the only example of linear evolution among the 15 trees. **(d) **Boxplots of the number of aberrations occurring at each step in tumor progression trees. CP, in the common precursor; PT, between the common precursor and the primary tumor; IR, between the common precursor and the ipsilateral recurrence. **P-value < 0.01, as determined in a two-tailed paired *t*-test.

Fifteen of the 22 pairs of primary breast carcinomas and ipsilateral recurrences studied by Bollet *et al. *[18] were shown to have a monoclonal origin. We analyzed these 15 pairs with the TuMult algorithm. A linear evolution was found in only one of the 15 pairs of tumors studied, pair 14 (Figure 5a), all the other pairs displaying events specific to the recurrence and events specific to the primary tumor (Figure 5b,c), consistent with the findings of Kuukasjärvi *et al. *[30] regarding primary tumors and metastases. A median of 17 aberrations occurred between the normal cell and the common precursor, 14 aberrations occurred between the common precursor and the primary tumor, and 26 aberrations occurred between the common precursor and the ipsilateral recurrence (Figure 5d). By contrast to what has been observed for bladder cancer, the number of aberrations specific to the recurrence was significantly higher than the number of aberrations specific to the primary tumor (*P *= 0.008). As all patients underwent radiotherapy and some also underwent chemotherapy between the primary tumor and the recurrence, it is unknown whether the higher complexity of the recurrences resulted from treatment or were intrinsic to the tumor progression process.

The 15 tumor progression trees were used to discriminate between early and late events in breast cancer development. We considered the 17 aberrations defined as frequent in breast carcinoma by Hwang *et al. *[31], determining the frequency of each of these aberrations on each edge of the trees. We then used a two-tailed Fisher's exact test to determine whether each aberration was associated with the early step (between the normal cell and the common precursor) or the late step (between the common precursor and the primary tumor) of tumor progression. The edge between the common precursor and the recurrence was not considered because some of the aberrations on this edge may have resulted from radiotherapy. Five events were found to be significantly associated with the early step: +1q, -6q, -8p, +8q, and -16q (Table 2). Consistent with these findings, +1q, -8p, +8q, and -16q were shown to be among the most frequent aberrations (≥35%) in ductal carcinoma *in situ*, a precursor of invasive breast carcinoma [31]. The other two aberrations also shown to be common in ductal carcinoma *in situ *by Hwang *et al*., -17p and +17q, were not identified as 'early' by our approach. However, our findings do not conflict with those of Hwang *et al. *for -17p, as this aberration was found in the common precursor in 40% of our trees, but was not considered to be significantly early because it also occurred in the late step in 20% of the trees. No alteration was found to be significantly more frequent in the late step, consistent with the conclusion of Hwang *et al. *that ductal carcinoma *in situ *is a genetically advanced lesion, with a degree of chromosome alteration similar to that in invasive breast cancers.

**Table 2:** Early or late occurrence of the most frequent aberrations in breast carcinoma

Aberration	Occurrence in the common precursor (CP)	Occurrence between the CP and the primary tumor	Association with early/late events
+1q	53%	13%	0.050^a^
-3p	7%	13%	1
-6q	40%	0%	0.017^a^
-8p	60%	0%	0.00070^c^
+8p	0%	7%	1
+8q	53%	0%	0.0022^b^
-9p	27%	0%	0.10
-11q	20%	7%	0.60
+11q	0%	7%	1
-14q	27%	7%	0.33
+16p	0%	7%	1
-16q	47%	7%	0.035^a^
-17p	40%	20%	0.43
+17q	20%	13%	1
-18p	7%	7%	1
-18q	27%	7%	0.33
+20q	0%	0%	1

^a^*P ≤ 0.05*; ^b^*P *< 0.01; ^c^*P *< 0.001; Fisher's exact test.

### Application to the study of metastatic progression in prostate cancer

In a recent article, Liu *et al. *[19] analyzed anatomically separate tumors from men who died from metastatic prostate cancer. They showed that although individual metastases displayed specific aberrations, all the samples from a given patient had a monoclonal origin, and maintained a signature copy number pattern of the precursor metastatic cancer cell. The Affymetrix Genome-Wide Human SNP Array 6.0 data from this article, comprising the copy number profiles of 58 metastatic samples taken from different anatomic sites in 14 patients, are available from the Gene Expression Omnibus database [32]. We used these data to reconstruct the tumor progression tree in each patient with the TuMult algorithm. Consistent with the conclusion of Liu *et al*., each tree displayed a common precursor of all samples, with a substantial number of aberrations (median = 26.5 events).

**Figure 6:**
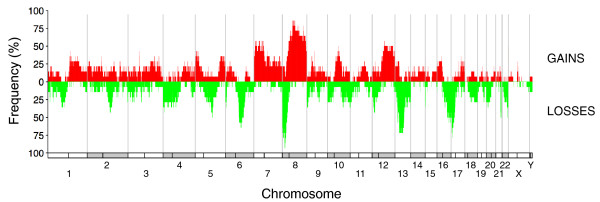
**Frequency of gains and losses in the metastatic precursor clones of 14 patients with metastatic prostate cancer**. Fourteen patients with various metastatic samples taken from different anatomic sites were analyzed with the TuMult algorithm to generate the lineage of the metastases and to reconstruct the copy number profile of the common precursor of all metastases in each patient. The frequency of gains (in red) and losses (in green) in the genome were calculated for these 14 metastatic precursor clones.

We first used the tumor progression trees to look for recurrent events at the onset of metastasis. In each tree, the common precursor of all metastases represents the ancestral clone from which all the metastases spread, and is thus likely to harbor the crucial alterations triggering metastasis. We determined the frequencies of gains and losses within the genome in the metastatic precursor clones of the 14 tumor progression trees (Figure 6). Thirteen aberrations were detected in more than half the precursors, including gains at 7p (57%), 8q (86%), 10q21 (50%), 12q (57%) and Xp22 (50%), and losses at 5q21 (50%), 6q14-21 (64%), 8p21 (93%), 13q13-22 (71%), 16q22-24 (57%), 17p13-11 (79%), 21q22 (50%) and 22q13 (50%). Loss of 8p21 (in 13 out of 14 cases) and gain of 8q24 (in 11 out of 14 cases) were the most frequent events in our metastatic precursor clones, suggesting that they may play a role in metastatic progression. Consistent with these observations are the findings that gain of MYC (8q24) is associated with poor prognosis in prostate cancer, and that the pattern of 8p21-22 loss with 8q24 gain is an independent risk factor for systemic progression and cancer-specific death in this disease [33].

**Figure 7:**
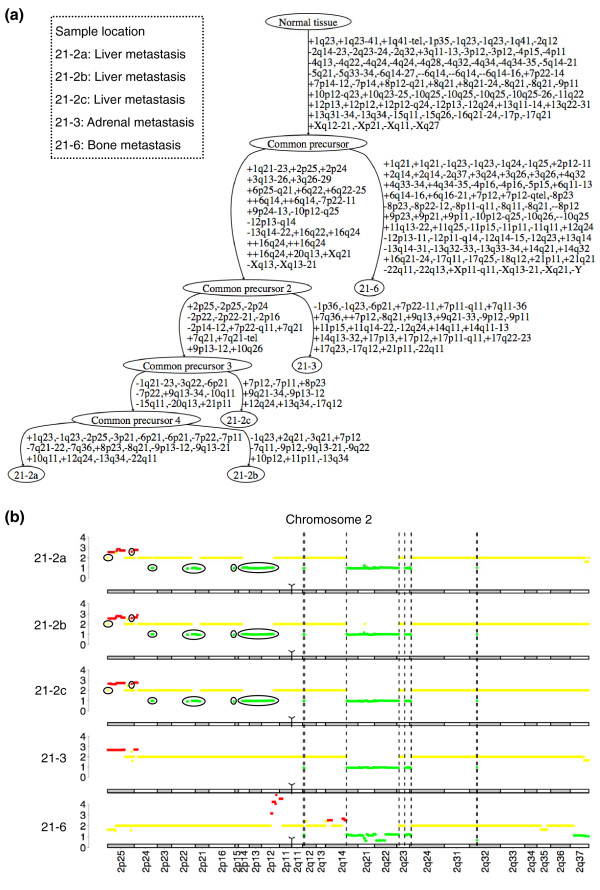
**Metastases spreading in a patient with prostate cancer**. Five metastases were removed from patient 21 (data from Liu *et al. *[19]), including three liver metastases (21-2a, 21-2b and 21-2c), one adrenal metastasis (21-3) and one bone metastasis (21-6). **(a) **Tumor progression tree reconstructed with the TuMult algorithm. The three liver metastases have a longer clonal relationship than the other metastases, all being derived from common precursor 3. **(b) **Copy number profiles of chromosome 2 in the five metastases. Four common aberrations, delimited by dashed lines, occurred in the common precursor of all the samples. The seven circled aberrations are specific to the three liver metastases, showing these tumors diverged later in the tumor progression tree. These aberrations appeared in common precursor 3, from which the liver metastases are derived.

For eight patients, the set of metastases included several metastases from the same organ, either at the same anatomic site (liver), or in the same type of organ, but at different locations (lymph nodes and bone metastases). We investigated whether metastases from a given organ were more closely related to each other in the trees than to metastases from other organs. The liver metastases were systematically more closely related to each other than to other metastases. They were always derived from a single precursor (as in patient 21; Figure 7a), with specific events not found in the other metastases (Figure 7b), forming a subtree in the tumor progression tree. This finding is significant, since the probability of observing such a pattern in the three patients by chance, calculated as the proportion of all the possible tree topologies in which liver metastases form a subtree, is only *P *= 0.003. By contrast, lymph node and bone metastases were often found together with other metastases in the tumor progression trees (Additional file 2). One possible interpretation of the late divergence of liver metastases is that specific alterations are required for liver invasion. Thus, all liver metastases would be likely to arise from a subclone of the prostate tumor with the required alterations. Alternatively, the invasion of the liver by one clone may be the limiting step for metastatic spread in this organ, with all the metastases in the liver resulting from the dissemination of a single clone successfully colonizing the organ. We favor this hypothesis because the alternative explanation would probably result in lymph node and bone metastases being closely related too, and because no organ-specific alterations were identified by Liu *et al*.

## Discussion

In this paper, we introduce a new method for unraveling the succession of chromosome aberrations occurring during the process of carcinogenesis. It has recently been shown that copy number data for several samples from the same patient can be used to demonstrate clonality [18], or to elucidate the biology underlying relapse [34] or metastasis [19]. However, a computational approach for automatically reconstructing tumor lineages and the sequence of chromosomal events from high-definition copy number data was lacking. Several algorithms for reconstructing trees from discrete character vectors or distance matrices have been developed in phylogenetics [20,35-39], but particular features specific to copy number data, in particular the cumulative nature of aberrations, made it necessary to develop a dedicated algorithm.

One of the key features of the TuMult algorithm is that it focuses on chromosome breakpoints, rather than aberrations, making it possible to reconstruct the ancestral chromosomal events from profiles with several imbricated aberrations. As a result, the performance of TuMult is little affected by the complexity of the trees, unlike the parsimony method, the performance of which declines rapidly with the occurrence of overlapping aberrations. However, reasoning in terms of breakpoints introduces additional difficulties. First, an odd number of common breakpoints may be identified for a given chromosome. This occurs in the rare cases in which a common breakpoint is erased by a subsequent breakpoint of the opposite sign at the same location, or when two independent aberrations share a common breakpoint by chance. In this case, some of the information required for inference of the sequence of events with certainty is lacking, so TuMult reconstructs the scenario involving the smallest number of changes. These rare situations occur mostly in conditions in which a large number of events have accumulated, accounting for the slight increase in error rates with increasing numbers of tumors. Second, the copy number profiles must be of sufficiently high quality for the identification of common breakpoints. With increasing noise and normal cell contamination, the breakpoints may be shifted a few probes away by segmentation algorithms. A tolerance threshold was introduced to deal with such samples. However, as increasing this threshold decreases the specificity of common breakpoints, we recommend the discarding of samples of very low quality (noise standard deviation > 0.12 or normal cell contamination > 50%) when analyzing data with TuMult. If these precautions are taken, the tumor progression trees reconstructed with TuMult are highly reliable, as demonstrated from our analysis of simulated data.

The applications of TuMult in cancer research are numerous. First, TuMult makes it possible to go back in time, reconstructing the genomic profiles of ancestral tumor clones of particular interest that are not accessible by sampling. We have shown that, in both bladder and breast cancers, recurrences do not generally arise directly from the primary tumor. The primary tumor thus displays many specific events and is poorly representative of the initial tumor progression step. By contrast, the common precursor of all samples usually displays a small set of aberrations, even if all the tumors studied have complex profiles. This strategy may be of particular interest when trying to identify the initial events in carcinogenesis, particularly for cancers in which early lesions are rarely accessible. Another ancestral clone of particular interest was highlighted when studying the metastatic process in prostate cancer. We were able to reconstruct the copy number alterations of the metastatic precursor clone giving rise to all the metastases in each patient. A few recurrent aberrations were identified in these clones. These aberrations potentially play an important role in metastatic spread and may be good predictors of metastasis in prostate cancer.

Second, tumor progression trees could be used in integrative studies aiming to develop a general model of tumor progression. In such studies, individual tumor progression trees are much more informative than a collection of individual samples, as the relative times at which aberrations occurred is already known in each patient. As a proof-of-concept, with only 15 breast tumor progression trees, we were able to identify most of the early aberrations previously described as characteristic of ductal breast carcinoma *in situ*, the precursor lesion of invasive breast cancers [31,40], despite our data corresponding only to invasive samples. Conversely, the analysis of cases with both superficial and invasive tumors may facilitate identification of the genes responsible for invasiveness, through studies of the aberrations occurring between the common precursors and invasive samples.

Finally, the tumor lineage *per se *may provide insight into the spread of cancerous cells to different parts of the organ, or different parts of the body. Analysis of the copy number profiles of metastases in the data set from the study by Liu *et al. *[19] revealed the relationships between the metastases in the different organs, showing that liver metastases always diverged from a single precursor clone.

One factor limiting the application of TuMult is the need to have access to several tumors from the same patient, either at different time points during the disease (recurrences), or from different sites at a single time point. Most tumors are thought to be heterogeneous, consisting of a mixture of clones that have diverged from the same initial cancer cell. The microdissection of distant parts of a single tumor may therefore make it possible to reconstruct the sequence of aberrations occurring during the clonal development of the tumor, providing access to early and late events, just like the analysis of samples from different tumors [41]. Using ultra-deep sequencing, Campbell *et al. *[42] were able to infer the interrelationships between subclones in two tumors. Most genomic analyses to date have focused on the search for similarities in large tumor data sets, with the aim of identifying the fundamental mechanisms of cancer. The next step may be to dig deeper into the dynamic pathways of cancer progression by analyzing the unique succession of changes driving carcinogenesis in each patient.

## Conclusions

We report here a novel computational approach for unraveling the successive copy number alterations driving carcinogenesis. We have shown that TuMult is highly reliable for reconstructing both tumor lineage and the copy number profiles of ancestral tumor clones, significantly outperforming the parsimony method. TuMult is a new tool of great interest for researchers seeking to develop a profound understanding of the development of cancer from the analysis of several samples. The annotated R code of the program is provided, together with detailed user instructions and examples of formatted data [43].

## Materials and methods

### Patients and bladder tumor samples

We obtained 28 bladder tumor samples from patients admitted between 1993 and 2002 to the Henri Mondor Hospital. Thirteen of these samples were multiple tumors from five patients (two to four metachronous or synchronous samples per patient; Table 1). The other 15 samples came from 15 independent patients (Additional file 3) and were used as the reference data set for estimation of the frequency of breakpoints at each location in the bladder SNP data. All subjects provided informed consent and the study was approved by the ethics committee of Henri Mondor Hospital. Flash-frozen tissue samples were stored at -80°C immediately after transurethral resection or cystectomy. DNA was extracted with the cesium chloride [44] or proteinase K/phenol/chloroform method. DNA purity was assessed by determining the ratio of absorbances at 260 and 280 nm. DNA concentration was determined with a Hoechst dye-based fluorescence assay [45].

### BAC array CGH

Eight bladder tumors from three patients were analyzed with HumArray 2.0 arrays, consisting of 2,385 BAC clones covering the human genome with an average resolution of 1.3 Mb, as previously described [46]. These arrays were obtained from the UCSF Cancer Center Array CGH Core Facility. Probes were labeled and hybridization was carried out as described elsewhere [47]. Images were analyzed with SPOT 2.0 software [48]. Poor-quality spots were removed in a pre-processing step: only spots with a reference signal intensity above 25% of the background reference signal (the 4,6-diamidino-2-phenylindole (DAPI) signal) were considered reliable. Spots located in zones of spatial bias were ignored [49]. No value was assigned to 25 BACs in the data, and these BACs were therefore eliminated from the analysis. Each BAC was assigned 'gain', 'normal' or 'loss' status, using the GLAD segmentation algorithm [50]. BACs with log2 ratios below -0.8 and over 0.8 were assigned 'homozygous deletion' and 'amplification' status, respectively.

### SNP arrays

We analyzed 20 bladder tumors, comprising 15 independent tumors and 5 multiple tumors from two patients, with Illumina HumanCNV370 Genotyping BeadChips (Illumina Inc.). Hybridization was performed by IntegraGen (Evry, France), according to the instructions provided by the array manufacturer. Fluorescent signals were imported into BeadStudio software (Illumina Inc.) and normalized. The normalized fluorescence signals for a sample were compared with the signal intensities of a set of reference genotypes in Beadstudio software, and the log2 ratios between the sample and reference signals (log R ratios (LRR)) were calculated as previously described [51]. In addition, the B-allele frequency (BAF) for the sample was estimated, based on the reference genotype clusters [51]. Segmentation was carried out with the BAFsegmentation algorithm [52,53], with amplitude (*ai.thr*) and size (*ai.size*) thresholds for significant allelic imbalance detection set at 0.53 and 10, respectively. As this method requires heterozygous SNPs, the × and Y chromosomes of the male patient were segmented based on LRR, using the CBS algorithm [54] (R package DNAcopy [55]) with default settings except for the significant level for accepting change-points (*α*), which was set to 0.001, and a size threshold of 10 SNPs for significant aberrations, which was added to ensure concordance with the smoothing for other chromosomes. A discrete copy number status was then assigned to each segment, based on its median LRR value. The zero level for normal copy number segments was first determined as the median LRR in regions of allelic balance, as previously decribed [56]. Segments with a median LRR more than 0.05 above the zero level were assigned 'gain' status and segments with a median LRR more than 0.5 above the zero level were assigned 'amplified' status. Segments with a median LRR more than 0.05 below the zero level were assigned 'loss' status and segments with a median LRR more than 0.5 below the zero level were assigned 'homozygous deletion' status. SNP data for breast samples were kindly provided by Bollet *et al*., and the details of array processing and copy number alteration determination for these samples are provided in the corresponding article [18].

### Generation of tumor lineage trees

#### Overview of the method

The clonal development of several tumors in a patient can be represented as a rooted tree, where the root represents the normal cell (*NC*), the leaves are the tumors removed from the patient, and the intermediate nodes are the common precursors of the observed samples. We want to reconstruct the most likely tumor lineage and to infer, simultaneously, the copy number profiles of the intermediate nodes. In other words, we want to reconstruct the copy number profiles of the common ancestors of the tumors. We use a bottom-up agglomerative strategy to do this. Let the *Front *be the set of nodes with no upstream edge at a given step of tree reconstruction. Initially, the *Front *is the set of tumors removed from the patient. At each step of the algorithm, the two closest nodes in the *Front*, in terms of breakpoints, are joined. The copy number profile of their common precursor is inferred, and the common precursor replaces the two nodes in the *Front*. This step is iterated until the *Front *contains only one node: the common ancestor of all samples.

#### Definition of breakpoint and amplitude vectors

We define a chromosome breakpoint as a change in copy number status between two consecutive probes. Chromosome breakpoints are also defined at chromosome ends, where copy number differs from the normal value. Before tumor lineage reconstruction, each chromosome is divided into 'homogeneous segments' delimited by all the breakpoints identified in the samples from the patient. A segment is thus a continuous set of probes for which copy number is constant in any sample from the patient. Let *N *be the number of segments delimited on the chromosome. For each sample, the copy number profile on the chromosome can be represented as a vector *s *ε {-2, -1, 0, 1, 2}^*N *^where *s*_*j *_represents the copy number of segment *j *(-2, homozygous deletion; -1, loss; 0, normal; 1, gain; 2, amplification). A breakpoint vector *b *is defined from *s *as follows:

$$\begin{array}{ll} b_{1}=s_{1} & \\ b_{i}=s_{i}-s_{i-1}, & 2\leq i\leq N\\ b_{N+1}=-s_{N} & \end{array}$$

There is therefore a breakpoint between segments *i *- 1 and *i *if, and only if, *b*_*i *_≠ 0. Each breakpoint is characterized by its sign: 'up' if *b*_*i *_> 0, 'down' if *b*_*i *_< 0, and its amplitude |*b*_*i*_|.

We need to consider 'up' and 'down' breakpoints separately. We have therefore simplified the notation, defining breakpoint amplitude vector *a *as follows:

$$\begin{array}{lll} a_{i}=b_{i} & if & b_{i}\geq 0\\ a_{N+1+i}=-b_{i} & if & b_{i}\leq 0 \end{array}$$

The amplitude vectors of all chromosomes are then joined end-to-end to form the amplitude vector of the whole profile. In the following, *a *refers to the amplitude vector of the whole profile of the tumor. The generation of the segment, breakpoint and amplitude vectors is illustrated in Additional file 4.

Note that the functions associating a segment vector *s *with a breakpoint vector *b *and a breakpoint vector *b *with an amplitude vector *a *are bijections. In other words, any amplitude vector corresponds to a unique breakpoint vector and a unique segment vector.

#### Biological viability of a chromosome breakpoint vector

All aberrations involve the occurrence of two breakpoints (one 'up' and one 'down'), so a breakpoint vector *b *represents a biologically viable chromosome only if 'up' and 'down' breakpoints are balanced:

$$\sum_{i=1}^{N+1}b_{i}=0$$

This condition is verified by definition in the tumor breakpoint vectors, but it must be checked for each chromosome when reconstructing the copy number profiles of common precursors.

#### Definition of a common breakpoint

We define a common breakpoint of tumors *T*_*i *_(represented by its breakpoint amplitude vector *a*^*i*^) and *T*_*j *_(represented by vector *a*^*j*^) as a breakpoint of the same sign observed at the same location in the two samples, that is, belonging to $\left\{k\;/a_{k}^{i}>0,\;a_{k}^{j}>0\right\}$. An adjustable tolerance threshold was introduced to recognize as common breakpoints, breakpoints located very close together, for which the slight difference in location probably resulted from small discrepancies in the smoothing process. For bladder samples, the 95th percentiles of the errors in the location of the breakpoints, estimated by permuting the markers within each segment and re-segmenting 100 times, were used as tolerance thresholds. The Bollet and Liu data were already discretized. We therefore used a threshold of ten probes for these data sets, consistent with the thresholds estimated for our bladder samples analyzed with SNP arrays.

#### Identical breakpoint score

At each step of the algorithm, the two nodes that separated most recently in the tumor lineage are joined. As chromosome aberrations accumulate during tumor development and are passed on to daughter cells, we can assume that the later two tumor clones separate, the more chromosome breakpoints they will have in common. We defined an identical breakpoint score (IBS) to quantify the number of breakpoints common to two nodes.

Let us consider two nodes, *N*_*i *_and *N*_*j*_, characterized by their amplitude vectors, *a*^*i *^and *a*^*j*^. We first define the common amplitude vector *a*^*ij *^as the amplitude vector of breakpoints common to nodes *N*_*i *_and *N*_*j*_:

$$a_{k}^{ij}=\min\left(a_{k}^{i},a_{k}^{j}\right)$$

As very frequent breakpoints are more likely than rare breakpoints to occur independently, by chance, on several occasions, the added value of each breakpoint is weighted down according to its frequency *F*_*k *_in a reference data set of independent tumors, as proposed by Bollet *et al. *[18]. Three data sets were used to estimate the frequencies of 'up' and 'down' breakpoints at each location: 50 previously published bladder tumors for BAC arrays [46], a set of 15 independent bladder tumors for SNP arrays, and the same set of 44 control samples used by Bollet *et al. *for breast tumors.

The IBS is simply the number of breakpoints common to the two nodes, weighted by their frequency in the reference data set:

$$IBS\left(a^{i},a^{j}\right)=\sum_{k}a_{k}^{ij}\left(1-F_{k}\right)$$

Similarly, we define the distance between two nodes as the number of breakpoints differing between the two profiles, weighted by their frequency in the reference data set:

$$D\left(a^{i},a^{j}\right)=\sum_{k}\left|a_{k}^{i}-a_{k}^{j}\right|\left(1-F_{k}\right)$$

At each step of the algorithm, the two nodes from the *Front *with the highest IBS are joined. If several pairs have the same IBS, the pair with the smallest distance is chosen.

#### Generation of the copy number profile of the common precursor of two samples

At each step of the algorithm, the profile of the common precursor of the two nodes joined, *CP*(*a*^*i*^,*a*^*j*^), is inferred. This profile is reconstructed chromosome by chromosome, through the identification of breakpoints common to the two nodes. In this paragraph, notations *a *and *b *therefore refer to the amplitude and breakpoint vectors of a single chromosome.

First, the common breakpoints are identified by computing the common amplitude vector of the two nodes, *a*^*ij*^, as described above:

$$a_{k}^{ij}=\min\left(a_{k}^{i},a_{k}^{j}\right)$$

The balance between the 'up' and 'down' breakpoints in this vector is then checked, because an unbalanced profile (for example, one common breakpoint 'up' and no common breakpoint 'down') does not define a viable copy number profile. This is done by inferring the common breakpoint vector *b*^*ij *^corresponding to *a*^*ij*^, and checking the balance between 'up' and 'down' breakpoints by calculating the sum *δ *of all the breakpoints affecting the chromosome:

$$\delta =\sum_{k}b_{k}^{ij}$$

Two situations emerge at this point. First, if *δ *= 0, 'up' and 'down' breakpoints are balanced, the amplitude vector of the common precursor is given directly by:

$$CP\left(a^{i},a^{j}\right)=a^{ij}$$

Second, if *δ *≠ 0, 'up' and 'down' breakpoints are unbalanced. The set of breakpoints in the common precursor does not define a viable copy number profile and requires correction (see next section) to restore the equilibrium.

#### Correction of unbalanced chromosomes

In rare cases, the number of common breakpoints of each sign ('up' and 'down') differ for a given chromosome:

$$\delta =\sum_{k}b_{k}^{ij}\neq 0$$

There are two possible reasons for this (Additional file 5). In the first case, if several aberrations appearing independently in several lineages have, by chance, a breakpoint in common, this breakpoint will be considered as a common breakpoint and incorporated into the common precursor without a counterpart of the opposite sign, leading to an unbalanced profile. In this case, the actual profile of the common precursor is obtained by removing the false common breakpoint. In the second case, if two aberrations successively affect two neighboring segments, a breakpoint may be erased by the occurrence of a breakpoint of the opposite sign at the same location. One of the breakpoints defining a common aberration may therefore be missing in one of the two descendant nodes, leading to an unbalanced profile. In this case, the actual profile of the common precursor is obtained by adding the breakpoint that has been erased to the profile of the common precursor.

Thus, to restore breakpoint equilibrium in the common precursor, we need to either remove a breakpoint of *sign*(*δ*) from the common breakpoint vector (the first case), or add a breakpoint of *sign*(-*δ*) chosen from the breakpoints specific to one of the two samples (the second case). As each choice generates a different common precursor and a different sequence of events for the chromosome considered, each possible modification is automatically evaluated in terms of the sequence of events it would generate. The breakpoint correction that does not lead to an impossible event (such as a segment deviating from the set of authorized values {-2, -1, 0, 1, 2}) and minimizes breakpoint usage is selected (Additional file 6), and the status of the breakpoint is corrected in the profile of the common precursor.

#### The particular case of amplicons

Amplicons are known to originate from mechanisms different from those responsible for generating losses and gains, including unscheduled DNA replication or inverted duplications [57]. In particular, amplicons cannot be considered as an accumulation of gains targeting the same region. We therefore treated amplicons separately from other aberrations. Once the copy number profile of the common precursor has been reconstructed, amplicons common to both descending nodes are incorporated into it, and amplicons specific to one of the descendant nodes are placed on the relevant edge.

### Visualization of the trees

The tumor progression trees produced by the TuMult algorithm were exported in .dot format. Images were produced from the .dot files with the open source graph visualization software Graphviz [58].

### Analysis of simulated tumor progression trees

#### Simulation of tumor progression trees

We implemented an algorithm to generate simulated tumor progression trees to validate the reconstruction methods. First, a common precursor is created from the 'Normal tissue' node. Then, at each step of the algorithm, a node is randomly chosen in the front, from which two new nodes are created, until the required number of tumors is obtained. The profile of each new node is determined as follows: 1, random selection of the number *n*_*ab *_of aberrations to add, between 3 and 15 (in line with the numbers observed in experimental trees); 2, random selection of *n*_*ab *_pairs of 'up' and 'down' breakpoints; 3, calculation of the copy number profile obtained by adding the aberrations thus defined to the previous node. Aberrations that would lead to an impossible copy number (out of {-2, -1, 0, 1 2}) are discarded and replaced by a new randomly generated aberration to avoid the creation of meaningless profiles. This algorithm was used to simulate tumor progression trees of random topologies, with various numbers of tumors. We obtained realistic copy number profiles by setting the number of probes to 2,360, as in our reference CGH data set for bladder tumors, and weighting the random sampling of breakpoints according to their frequency in this reference data set.

#### Effects of noise and normal cell contamination

We investigated the impact of noise in the log ratio and normal cell contamination on the performance of the method by generating simulated log ratio signals from the discrete copy number profiles of each node. For each copy number status *N*, the theoretical log ratio value was calculated as a function of the percentage *C *of normal cells in the sample:

$$\log ratio=\log\left(\frac{N\times \left(1-C\right)+2\times C}{2}\right)$$

Noise was then simulated by adding random values drawn from a normal distribution with a mean of 0 and a standard deviation of *S*. Simulations were carried out for values of *C *from 0 to 0.8 (80% of normal cells in the sample), and values of *S *from 0 to 0.2. The values of *S *in our experimental data, estimated as the standard deviation of the difference between raw and smoothed log ratios after segmentation, were between 0.03 and 0.11. The percentage *C *of normal cells in each sample was between 10 and 40%, as assessed by hematoxylin and eosin staining of the histological sections adjacent to those analyzed.

#### Reconstructing tumor progression trees with the parsimony method

The parsimony method [39] identifies the tree with the minimum number of changes from a set of leaves characterized by discrete character vectors. It could therefore be used to benchmark the TuMult algorithm. For this purpose, each tumor profile was divided into *N *'homogeneous segments', as in the preliminary step for the TuMult algorithm, and represented as a vector *s *ε {-2, -1, 0, 1, 2}^*N *^where *s*_*j *_is the copy number of segment *j*. These discrete vectors were used as an input for the *pars *program of the Phylip package [20].

#### Evaluation of the reconstructed tree

We evaluated the two methods by comparing each reconstructed tree with the original simulated tree. We first checked that the topology of the reconstructed tree, and hence the inferred tumor lineage, was identical to that in the simulated tree. We did this by calculating the Robinson-Foulds distance [59] between the two trees with the *treedist *program in the Phylip package [20]. A distance of zero indicates that the trees are identical. For each simulation data set, we calculated the percentage of the trees correctly inferred by each algorithm (Robinson-Foulds distance = 0). Then, for the trees with the correct topology, we checked that the copy number profiles of the internal nodes (corresponding to ancestral clones) were correctly reconstructed. We did this by calculating, for each reconstructed tree, the proportion of probes per node with a discrete copy number status different from that in the original simulated tree.

### Determination of co-occurring events in bladder carcinogenesis

If two aberrations are found on the same edge of a tumor progression tree, they must have occurred in the same time interval. Therefore, if two aberrations have a strong synergistic effect on tumor growth, they would be expected to be found on the same edge of a tumor progression tree significantly more frequently than would be expected by chance. We defined a set of aberrations frequently observed in bladder cancer on the basis of their frequency in the reference data set of 50 samples. Gains and losses were considered to be frequent in bladder cancer if they occurred in more than 20% of samples, whereas amplicons and homozygous deletions were considered frequent if they occurred in more than 10% of samples. Only aberrations found in at least three edges in the tumor progression trees were retained for further analysis. The co-occurrence of each pair of aberrations was then assessed by Fisher's exact tests, with correction for multiple testing, as previously described [60], using the q-value package in the R computing environment. The false discovery rate was set at 15%.

### Identification of early and late events in breast carcinogenesis through the integration of individual tumor progression trees

The tumor progression trees obtained from 15 pairs of primary breast carcinomas and ipsilateral recurrences were used to discriminate between early and late events in this disease. These trees contain three edges: one edge from the normal cell to the common precusor (CP), one edge from the CP to the primary tumor, and one edge from the CP to the recurrence. We therefore defined an aberration as early if it occurred between the normal cell and the CP, and as late if it occurred between the CP and the primary tumor. Aberrations occurring between the CP and the recurrence were not considered because they may have resulted from radiotherapy administered between the primary tumor and the recurrence. The 17 chromosomal aberrations identified as common in breast carcinoma by Hwang *et al. *[31] were analyzed. We first calculated the frequency of each aberration as an early or late step in the 15 tumor progression trees. As these aberrations span whole chromosome arms, we considered an aberration to be present on an edge if 60% of the probes on this arm were aberrant. Finally, we used two-tailed Fisher's exact tests to determine whether each aberration was significantly more present in the early or late step of carcinogenesis.

### Availability

The TuMult algorithm was implemented in the R language. The annotated code is provided, together with detailed instructions for use and examples of formatted data [43]. CGH and SNP data for the 78 bladder tumors used in this study (CGH data comprises 8 multiple tumors and the reference data set of 50 samples; SNP data comprises 5 multiple tumors and the reference data set of 15 samples) are available through Gene Expression Omnibus [32] accession number [GEO: GSE19195].

## Abbreviations

BAC: bacterial artificial chromosome; CGH: comparative genomic hybridization; CP: common precursor; IBS: identical breakpoint score; SNP: single nucleotide polymorphism.

## Authors' contributions

EL, FR, FG and YA came up with the idea for this study. EL developed and implemented the method under the supervision of FG. YA provided bladder samples and clinical information. MB provided breast cancer data. All authors participated in the evaluation of the results. EL wrote the manuscript under the supervision of FG and FR. All of the authors have read and approved the final manuscript.

## Supplementary Material

Additional file 1 **Consistency of tumor progression trees and the partial identity score for sample clonality**. The partial identity score was developed by Bollet *et al. *[18] to determine whether two samples have a monoclonal origin. We investigated the consistency of our results with this approach by investigating the clonality of our bladder samples with the partial identity score (top), and reconstructing tumor progression trees for the pairs of breast samples characterized as non-clonal in the paper by Bollet *et al. *(bottom). **(a) **The partial identity scores were calculated for each pair of bladder tumors analyzed. The distributions of these scores for pairs of samples from different patients were calculated from the reference data sets (left, BAC array data; right, SNP data). The 95% quantile, used by Bollet *et al. *as the threshold for classifying a pair as monoclonal, is indicated as a red line. The only pairs of samples from the same patient with a partial identity score below the threshold were those involving sample S5C. Detailed numbering of pairs for CGH data: 1, S1A-S1B; 2, S2A-S2B; 3, S3A-S3D; 4, S3B-S3D; 5, S3C-S3D; 6, S3A-S3B; 7, S3A-S3C; 8, S3B-S3C. Detailed numbering of pairs for SNP data: 1, S4A-S4B; 2, S5A-S5B; 3, S5A-S5C; 4, S5B-S5C. **(b) **The tumor progression tree obtained for the pair of breast tumors P2, classified as non-clonal by Bollet *et al. *TuMult identified no common events between the samples. **(c) **Boxplots of the number of aberrations occurring at each step in the tumor progression trees obtained for true recurrences (left) or new primary tumors (right). CP, in the common precursor; PT, between the common precursor and the primary tumor; IR, between the common precursor and the ipsilateral recurrence. Very few events are found in the common precursors of the trees for new primary tumors, consistent with their low partial identity scores. Click here for file

Additional file 2 **Tumor progression trees of metastatic prostate cancers with several metastases from the same anatomic site or type of organ**. **(a) **Tumor progression trees of three patients with several metastases from the same anatomic site (liver). Liver samples were always more closely related to each other than to metastases from the other organs. In each tree, the liver samples are derived from a single common precursor, with a substantial number of events not encountered in the other samples. **(b) **Tumor progression trees of six patients with several metastases from the same type of organ but at different anatomic sites (lymph node and/or bone metastases). The tumors from the same type of organ are associated in P24, P28 and P30, but not in P17, P32 and P33. Click here for file

Additional file 3 **Clinical data for the 15 bladder samples constituting the reference data set for bladder SNP data**. Click here for file

Additional file 4 **Segmentation of the profiles and generation of the amplitude vectors**. Generation of the amplitude matrix. **(a) **Discretized copy number profiles of three tumors for a given chromosome (yellow, 'normal copy number'; green, 'loss'; red, 'gain'). The four breakpoints identified in the samples (dashed lines) divide the chromosome into five 'homogeneous segments'. **(b) **The profiles are equally well represented in a segment matrix, in which the copy number for each segment and each sample is encoded by an integer (-1, loss; 0, normal; 1, gain), in a breakpoint matrix, in which each value represents the difference in copy number between two adjacent segments, or an amplitude matrix, in which 'up' and 'down' breakpoints are distinguished by their position in the vector. A common breakpoint (gray regions) appears as a number of the same sign at the same position in the breakpoint matrix, or as a non-zero number at the same position in the amplitude matrix. Click here for file

Additional file 5 **Two scenarios lead to an unbalanced chromosome in the common precursor**. **(a) **Left: the two tumors independently acquire two different aberrations with a breakpoint in common. Right: the 'up' breakpoint between segments B and C in the common precursor is lost in tumor 2 due to the loss of the neighboring segment C. **(b) **In both cases, only one breakpoint remains common to both samples, resulting in an unbalanced chromosome for their common precursor. Click here for file

Additional file 6 **Correction of an unbalanced chromosome**. Detailed procedure of the correction of chromosomes with unbalanced 'up' and 'down' breakpoints. Click here for file
