# Supplementary figures and images for: Analysis of the copy number profiles of several tumor samples from the same patient reveals the successive steps in tumorigenesis
Source: Genome Biol. 2010 Jul 22;11(7):R76. doi: 10.1186/gb-2010-11-7-r76 (PMC2926787; doi:10.1186/gb-2010-11-7-r76)

(a)

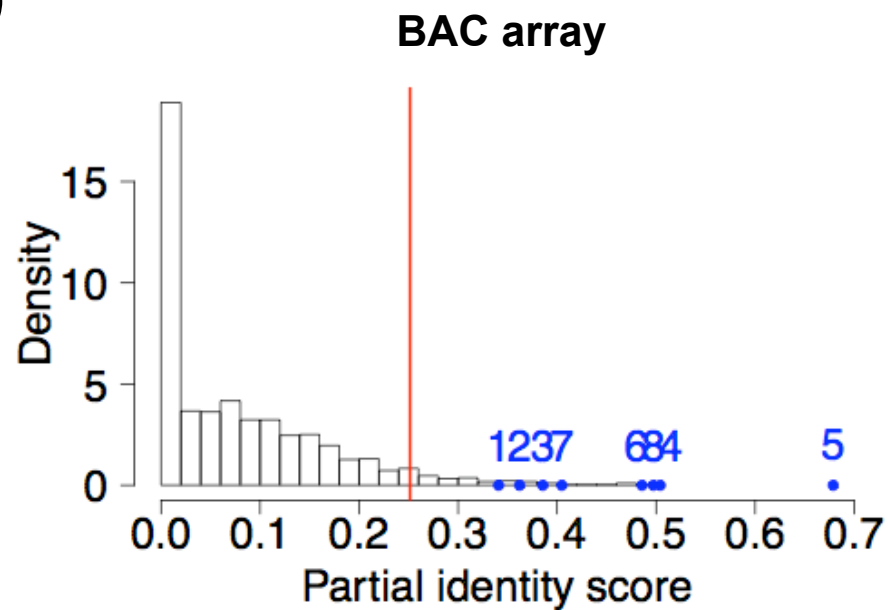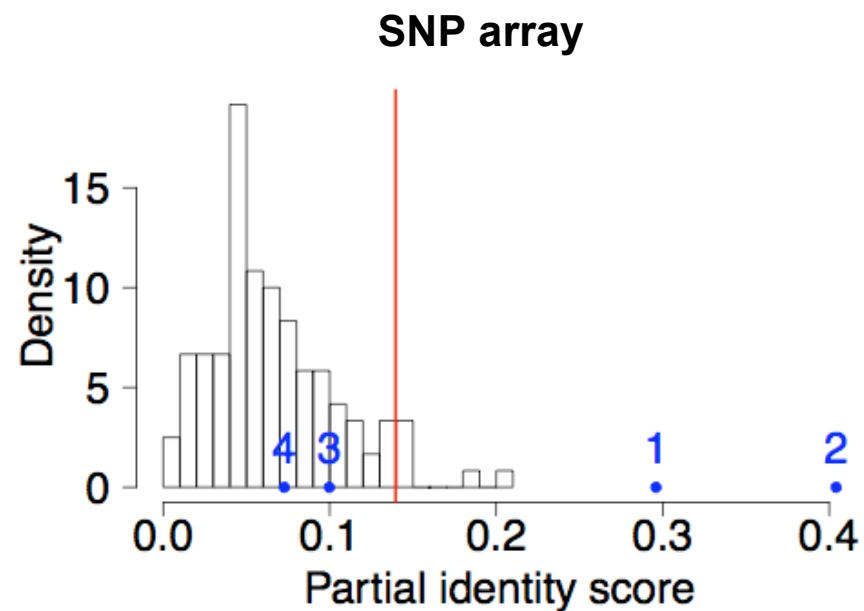

(b)

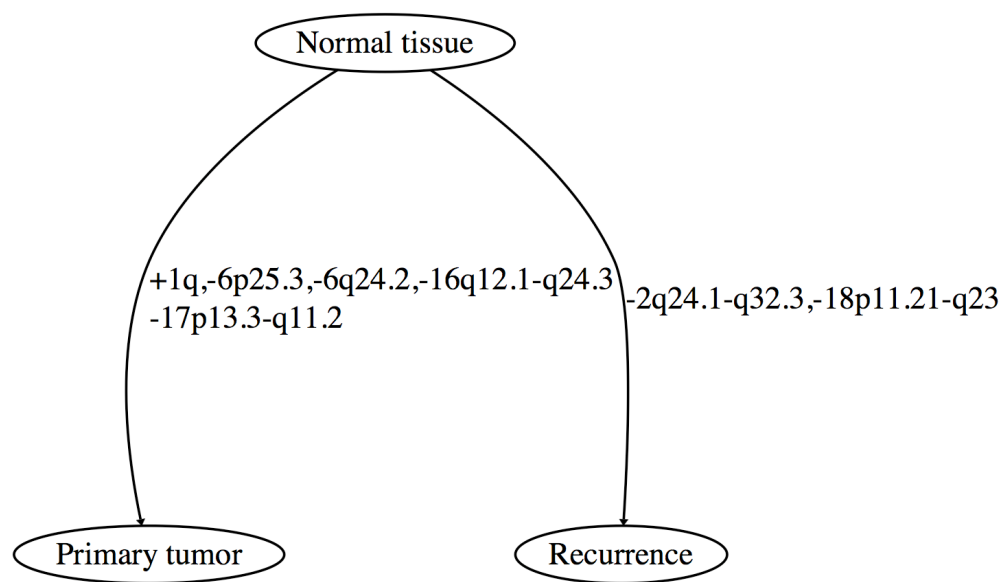

(c)

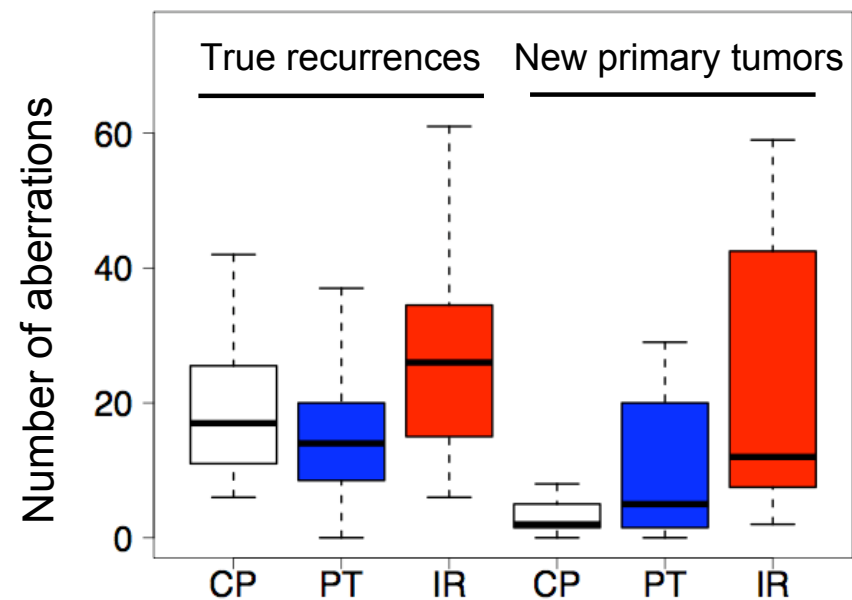

Supplement: Additional file 1 — Consistency of tumor progression trees and the partial identity score for sample clonality. The partial identity score was developed by Bollet et al. [18] to determine whether two samples have a monoclonal origin. We investigated the consistency of our results with this approach by investigating the clonality of our bladder samples with the partial identity score (top), and reconstructing tumor progression trees for the pairs of breast samples characterized as non-clonal in the paper by Bollet et al. (bottom). (a) The partial identity scores were calculated for each pair of bladder tumors analyzed. The distributions of these scores for pairs of samples from different patients were calculated from the reference data sets (left, BAC array data; right, SNP data). The 95% quantile, used by Bollet et al. as the threshold for classifying a pair as monoclonal, is indicated as a red line. The only pairs of samples from the same patient with a partial identity score below the threshold were those involving sample S5C. Detailed numbering of pairs for CGH data: 1, S1A-S1B; 2, S2A-S2B; 3, S3A-S3D; 4, S3B-S3D; 5, S3C-S3D; 6, S3A-S3B; 7, S3A-S3C; 8, S3B-S3C. Detailed numbering of pairs for SNP data: 1, S4A-S4B; 2, S5A-S5B; 3, S5A-S5C; 4, S5B-S5C. (b) The tumor progression tree obtained for the pair of breast tumors P2, classified as non-clonal by Bollet et al. TuMult identified no common events between the samples. (c) Boxplots of the number of aberrations occurring at each step in the tumor progression trees obtained for true recurrences (left) or new primary tumors (right). CP, in the common precursor; PT, between the common precursor and the primary tumor; IR, between the common precursor and the ipsilateral recurrence. Very few events are found in the common precursors of the trees for new primary tumors, consistent with their low partial identity scores. [file gb-2010-11-7-r76-S1.PDF]
