# Supplementary material for: Analysis of the copy number profiles of several tumor samples from the same patient reveals the successive steps in tumorigenesis
Source: Genome Biol. 2010 Jul 22;11(7):R76. doi: 10.1186/gb-2010-11-7-r76 (PMC2926787; doi:10.1186/gb-2010-11-7-r76)
Supplement: Additional file 2 — Tumor progression trees of metastatic prostate cancers with several metastases from the same anatomic site or type of organ. (a) Tumor progression trees of three patients with several metastases from the same anatomic site (liver). Liver samples were always more closely related to each other than to metastases from the other organs. In each tree, the liver samples are derived from a single common precursor, with a substantial number of events not encountered in the other samples. (b) Tumor progression trees of six patients with several metastases from the same type of organ but at different anatomic sites (lymph node and/or bone metastases). The tumors from the same type of organ are associated in P24, P28 and P30, but not in P17, P32 and P33. [file gb-2010-11-7-r76-S2.PDF]

(a)

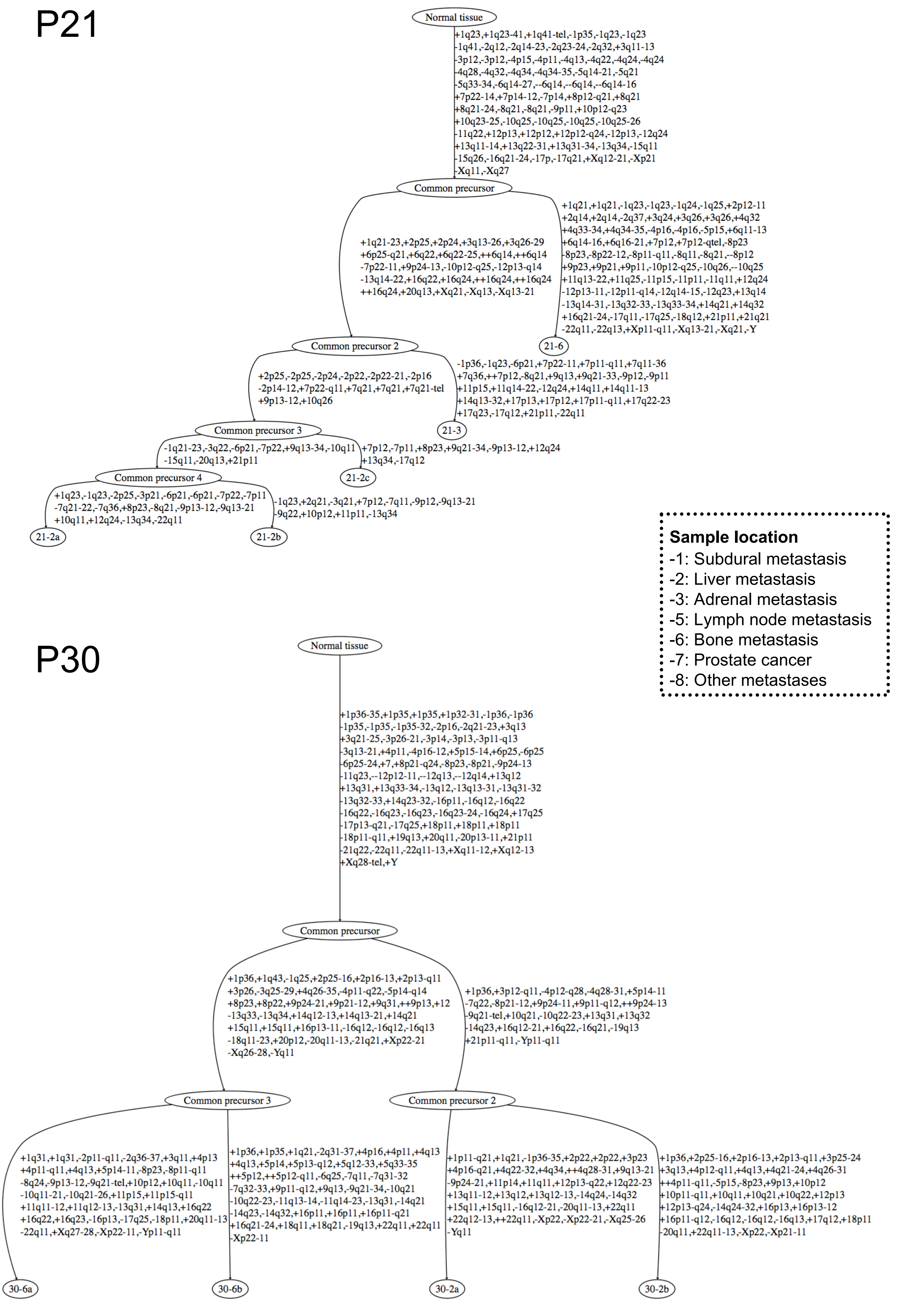
P21
Normal tissue
+1q23,+1q23-41,+1q41-tel,-1p35,-1q23,-1q23
-1q41,-2q12,-2q14-23,-2q23-24,-2q32,+3q11-13
-3p12,-3p12,-4p15,-4p11,-4q13,-4q22,-4q24,-4q24
-4q28,-4q32,-4q34,-4q34-35,-5q14-21,-5q21
-5q33-34,-6q14-27,--6q14,--6q14,--6q14-16
+7p22-14,+7p14-12,-7p14,+8p12-q21,+8q21
+8q21-24,-8q21,-8q21,-9p11,+10p12-q23
+10q23-25,-10q25,-10q25,-10q25,-10q25-26
-11q22,+12p13,+12p12,+12p12-q24,-12p13,-12q24
+13q11-14,+13q22-31,+13q31-34,-13q34,-15q11
-15q26,-16q21-24,-17p,-17q21,+Xq12-21,-Xp21
-Xq11,-Xq27
Common precursor
+1q21-23,+2p25,+2p24,+3q13-26,+3q26-29
+6p25-q21,+6q22,+6q22-25,++6q14,++6q14
-7p22-11,+9p24-13,-10p12-q25,-12p13-q14
-13q14-22,+16q22,+16q24,++16q24,++16q24
++16q24,+20q13,+Xq21,-Xq13,-Xq13-21
+1q21,+1q21,-1q23,-1q23,-1q24,-1q25,+2p12-11
+2q14,+2q14,-2q37,+3q24,+3q26,+3q26,+4q32
+4q33-34,+4q34-35,-4p16,-4p16,-5p15,+6q11-13
+6q14-16,+6q16-21,+7p12,+7p12-qtel,-8p23
-8p23,-8p22-12,-8p11-q11,-8q11,-8q21,--8p12
+9p23,+9p21,+9p11,-10p12-q25,-10q26,--10q25
+11q13-22,+11q25,-11p15,-11p11,-11q11,+12q24
-12p13-11,-12p11-q14,-12q14-15,-12q23,+13q14
-13q14-31,-13q32-33,-13q33-34,+14q21,+14q32
+16q21-24,-17q11,-17q25,-18q12,+21p11,+21q21
-22q11,-22q13,+Xp11-q11,-Xq13-21,-Xq21,-Y
21-6
Common precursor 2
+2p25,-2p25,-2p24,-2p22,-2p22-21,-2p16
-2p14-12,+7p22-q11,+7q21,+7q21,+7q21-tel
+9p13-12,+10q26
-1p36,-1q23,-6p21,+7p22-11,+7p11-q11,+7q11-36
+7q36,++7p12,-8q21,+9q13,+9q21-33,-9p12,-9p11
+11p15,+11q14-22,-12q24,+14q11,+14q11-13
+14q13-32,+17p13,+17p12,+17p11-q11,+17q22-23
+17q23,-17q12,+21p11,-22q11
21-3
Common precursor 3
-1q21-23,-3q22,-6p21,-7p22,+9q13-34,-10q11
-15q11,-20q13,+21p11
+7p12,-7p11,+8p23,+9q21-34,-9p13-12,+12q24
+13q34,-17q12
21-2c
Common precursor 4
+1q23,-1q23,-2p25,-3p21,-6p21,-6p21,-7p22,-7p11
-7q21-22,-7q36,+8p23,-8q21,-9p13-12,-9q13-21
+10q11,+12q24,-13q34,-22q11
-1q23,+2q21,-3q21,+7p12,-7q11,-9p12,-9q13-21
-9q22,+10p12,+11p11,-13q34
21-2a
21-2b
Sample location
-1: Subdural metastasis
-2: Liver metastasis
-3: Adrenal metastasis
-5: Lymph node metastasis
-6: Bone metastasis
-7: Prostate cancer
-8: Other metastases
P30
Normal tissue
+1p36-35,+1p35,+1p35,+1p32-31,-1p36,-1p36
-1p35,-1p35,-1p35-32,-2p16,-2q21-23,+3q13
+3q21-25,-3p26-21,-3p14,-3p13,-3p11-q13
-3q13-21,+4p11,-4p16-12,+5p15-14,+6p25,-6p25
-6p25-24,+7,+8p21-q24,-8p23,-8p21,-9p24-13
-11q23,--12p12-11,--12q13,--12q14,+13q12
+13q31,+13q33-34,-13q12,-13q13-31,-13q31-32
-13q32-33,+14q23-32,-16p11,-16q12,-16q22
-16q22,-16q23,-16q23,-16q23-24,-16q24,+17q25
-17p13-q21,-17q25,+18p11,+18p11,+18p11
-18p11-q11,+19q13,+20q11,-20p13-11,+21p11
-21q22,-22q11,-22q11-13,+Xq11-12,+Xq12-13
+Xq28-tel,+Y
Common precursor
+1p36,+1q43,-1q25,+2p25-16,+2p16-13,+2p13-q11
+3p26,-3q25-29,+4q26-35,-4p11-q22,-5p14-q14
+8p23,+8p22,+9p24-21,+9p21-12,+9q31,+9p13,+12
-13q33,-13q34,+14q12-13,+14q13-21,+14q21
+15q11,+15q11,+16p13-11,-16q12,-16q12,-16q13
-18q11-23,+20p12,-20q11-13,-21q21,+Xp22-21
-Xq26-28,-Yq11
+1p36,+3p12-q11,-4p12-q28,-4q28-31,+5p14-11
-7q22,-8p21-12,+9p24-11,+9p11-q12,++9p24-13
-9q21-tel,+10q21,-10q22-23,+13q31,+13q32
-14q23,+16p12-21,+16q22,-16q21,-19q13
+21p11-q11,-Yp11-q11
Common precursor 3
Common precursor 2
+1q31,+1q31,-2p11-q11,-2q36-37,+3q11,+4p13
+4p11-q11,+4q13,+5p14-11,-8p23,-8p11-q11
-8q24,-9p13-12,-9q21-tel,+10p12,+10q11,-10q11
-10q11-21,-10q21-26,+11p15,+11p15-q11
+11q11-12,+11q12-13,-13q31,+14q13,+16q22
+16q22,+16q23,-16p13,-17q25,-18p11,+20q11-13
-22q11,+Xq27-28,-Xp22-11,-Yp11-q11
+1p36,+1p35,+1q21,-2q31-37,+4p16,+4p11,+4q13
+4q13,+5p14,+5p13-q12,+5q12-33,+5q33-35
++5p12,++5p12-q11,-6q25,-7q11,-7q31-32
-7q32-33,+9p11-q12,+9q13,-9q21-34,-10q21
-10q22-23,-11q13-14,-11q14-23,-13q31,-14q21
-14q23,-14q32,+16p11,+16p11,+16p11-q21
+16q21-24,+18q11,+18q21,-19q13,+22q11,+22q11
-Xp22-11
+1p11-q21,+1q21,-1p36-35,+2p22,+2p22,+3p23
+4p16-q21,+4q22-32,+4q34,++4q28-31,+9q13-21
-9p24-21,+11p14,+11q11,+12p13-q22,+12q22-23
+13q11-12,+13q12,+13q12-13,-14q24,-14q32
+15q11,+15q11,-16q12-21,-20q11-13,+22q11
+22q12-13,++22q11,-Xp22,-Xp22-21,-Xq25-26
-Yq11
+1p36,+2p25-16,+2p16-13,+2p13-q11,+3p25-24
+3q13,+4p12-q11,+4q13,+4q21-24,+4q26-31
++4p11-q11,-5p15,-8p23,+9p13,+10p12
+10p11-q11,+10q11,+10q21,+10q22,+12p13
+12p13-q24,-14q24-32,+16p13,+16p13-12
+16p11-q12,-16q12,-16q12,-16q13,+17q12,+18p11
-20q11,+22q11-13,-Xp22,-Xp21-11
30-6a
30-6b
30-2a
30-2b

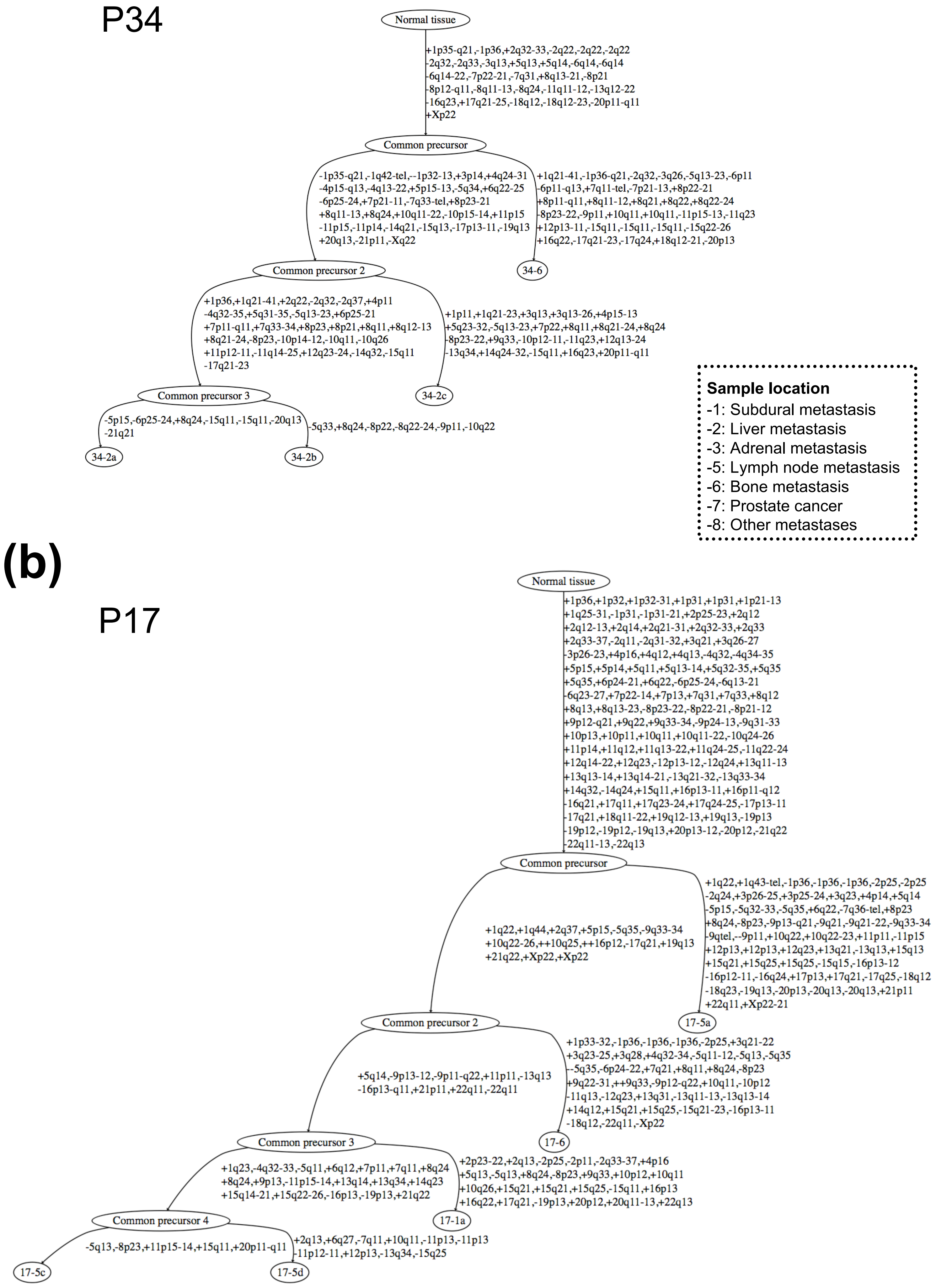
P34
Normal tissue
+1p35-q21,-1p36,+2q32-33,-2q22,-2q22,-2q22
-2q32,-2q33,-3q13,+5q13,+5q14,-6q14,-6q14
-6q14-22,-7p22-21,-7q31,+8q13-21,-8p21
-8p12-q11,-8q11-13,-8q24,-11q11-12,-13q12-22
-16q23,+17q21-25,-18q12,-18q12-23,-20p11-q11
+Xp22
Common precursor
-1p35-q21,-1q42-tel,--1p32-13,+3p14,+4q24-31
-4p15-q13,-4q13-22,+5p15-13,-5q34,+6q22-25
-6p25-24,+7p21-11,-7q33-tel,+8p23-21
+8q11-13,+8q24,+10q11-22,-10p15-14,+11p15
-11p15,-11p14,-14q21,-15q13,-17p13-11,-19q13
+20q13,-21p11,-Xq22
+1q21-41,-1p36-q21,-2q32,-3q26,-5q13-23,-6p11
-6p11-q13,+7q11-tel,-7p21-13,+8p22-21
+8p11-q11,+8q11-12,+8q21,+8q22,+8q22-24
-8p23-22,-9p11,+10q11,+10q11,-11p15-13,-11q23
+12p13-11,-15q11,-15q11,-15q11,-15q22-26
+16q22,-17q21-23,-17q24,+18q12-21,-20p13
Common precursor 2
34-6
+1p36,+1q21-41,+2q22,-2q32,-2q37,+4p11
-4q32-35,+5q31-35,-5q13-23,+6p25-21
+7p11-q11,+7q33-34,+8p23,+8p21,+8q11,+8q12-13
+8q21-24,-8p23,-10p14-12,-10q11,-10q26
+11p12-11,-11q14-25,+12q23-24,-14q32,-15q11
-17q21-23
+1p11,+1q21-23,+3q13,+3q13-26,+4p15-13
+5q23-32,-5q13-23,+7p22,+8q11,+8q21-24,+8q24
-8p23-22,+9q33,-10p12-11,-11q23,+12q13-24
-13q34,+14q24-32,-15q11,+16q23,+20p11-q11
Common precursor 3
34-2c
-5p15,-6p25-24,+8q24,-15q11,-15q11,-20q13
-21q21
-5q33,+8q24,-8p22,-8q22-24,-9p11,-10q22
34-2a
34-2b
Sample location
-1: Subdural metastasis
-2: Liver metastasis
-3: Adrenal metastasis
-5: Lymph node metastasis
-6: Bone metastasis
-7: Prostate cancer
-8: Other metastases
(b)
P17
Normal tissue
+1p36,+1p32,+1p32-31,+1p31,+1p31,+1p21-13
+1q25-31,-1p31,-1p31-21,+2p25-23,+2q12
+2q12-13,+2q14,+2q21-31,+2q32-33,+2q33
+2q33-37,-2q11,-2q31-32,+3q21,+3q26-27
-3p26-23,+4p16,+4q12,+4q13,-4q32,-4q34-35
+5p15,+5p14,+5q11,+5q13-14,+5q32-35,+5q35
+5q35,+6p24-21,+6q22,-6p25-24,-6q13-21
-6q23-27,+7p22-14,+7p13,+7q31,+7q33,+8q12
+8q13,+8q13-23,-8p23-22,-8p22-21,-8p21-12
+9p12-q21,+9q22,+9q33-34,-9p24-13,-9q31-33
+10p13,+10p11,+10q11,+10q11-22,-10q24-26
+11p14,+11q12,+11q13-22,+11q24-25,-11q22-24
+12q14-22,+12q23,-12p13-12,-12q24,+13q11-13
+13q13-14,+13q14-21,-13q21-32,-13q33-34
+14q32,-14q24,+15q11,+16p13-11,+16p11-q12
-16q21,+17q11,+17q23-24,+17q24-25,-17p13-11
-17q21,+18q11-22,+19q12-13,+19q13,-19p13
-19p12,-19p12,-19q13,+20p13-12,-20p12,-21q22
-22q11-13,-22q13
Common precursor
+1q22,+1q43-tel,-1p36,-1p36,-1p36,-2p25,-2p25
-2q24,+3p26-25,+3p25-24,+3q23,+4p14,+5q14
-5p15,-5q32-33,-5q35,+6q22,-7q36-tel,+8p23
+8q24,-8p23,-9p13-q21,-9q21,-9q21-22,-9q33-34
-9qtel,--9p11,+10q22,+10q22-23,+11p11,-11p15
+12p13,+12p13,+12q23,+13q21,-13q13,+15q13
+15q21,+15q25,+15q25,-15q15,-16p13-12
-16p12-11,-16q24,+17p13,+17q21,-17q25,-18q12
-18q23,-19q13,-20p13,-20q13,-20q13,+21p11
+22q11,+Xp22-21
+1q22,+1q44,+2q37,+5p15,-5q35,-9q33-34
+10q22-26,++10q25,++16p12,-17q21,+19q13
+21q22,+Xp22,+Xp22
Common precursor 2
17-5a
+1p33-32,-1p36,-1p36,-2p25,+3q21-22
+3q23-25,+3q28,+4q32-34,-5q11-12,-5q13,-5q35
--5q35,-6p24-22,+7q21,+8q11,+8q24,-8p23
+9q22-31,+9q33,-9p12-q22,+10q11,-10p12
-11q13,-12q23,+13q31,-13q11-13,-13q13-14
+14q12,+15q21,+15q25,-15q21-23,-16p13-11
-18q12,-22q11,-Xp22
+5q14,-9p13-12,-9p11-q22,+11p11,-13q13
-16p13-q11,+21p11,+22q11,-22q11
Common precursor 3
17-6
+1q23,-4q32-33,-5q11,+6q12,+7p11,+7q11,+8q24
+8q24,+9p13,-11p15-14,+13q14,+13q34,+14q23
+15q14-21,+15q22-26,-16p13,-19p13,+21q22
+2p23-22,+2q13,-2p25,-2p11,-2q33-37,+4p16
+5q13,-5q13,+8q24,-8p23,+9q33,+10p12,+10q11
+10q26,+15q21,+15q21,+15q25,-15q11,+16p13
+16q22,+17q21,-19p13,+20p12,+20q11-13,+22q13
Common precursor 4
17-1a
-5q13,-8p23,+11p15-14,+15q11,+20p11-q11
+2q13,+6q27,-7q11,+10q11,-11p13,-11p13
-11p12-11,+12p13,-13q34,-15q25
17-5c
17-5d

P24

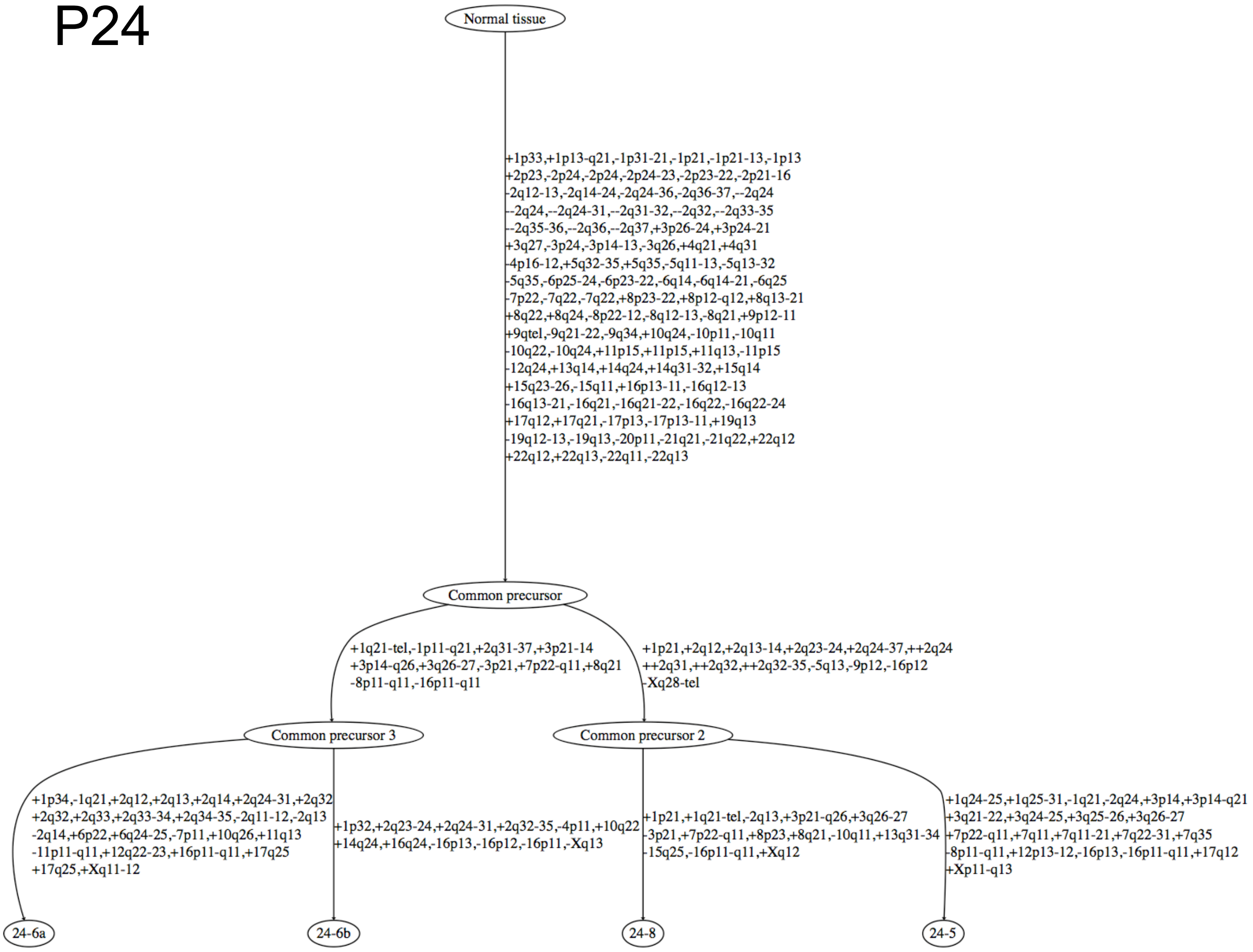
Normal tissue
+1p33,+1p13-q21,-1p31-21,-1p21,-1p21-13,-1p13
+2p23,-2p24,-2p24,-2p24-23,-2p23-22,-2p21-16
-2q12-13,-2q14-24,-2q24-36,-2q36-37,--2q24
--2q24,--2q24-31,--2q31-32,--2q32,--2q33-35
--2q35-36,--2q36,--2q37,+3p26-24,+3p24-21
+3q27,-3p24,-3p14-13,-3q26,+4q21,+4q31
-4p16-12,+5q32-35,+5q35,-5q11-13,-5q13-32
-5q35,-6p25-24,-6p23-22,-6q14,-6q14-21,-6q25
-7p22,-7q22,-7q22,+8p23-22,+8p12-q12,+8q13-21
+8q22,+8q24,-8p22-12,-8q12-13,-8q21,+9p12-11
+9qtel,-9q21-22,-9q34,+10q24,-10p11,-10q11
-10q22,-10q24,+11p15,+11p15,+11q13,-11p15
-12q24,+13q14,+14q24,+14q31-32,+15q14
+15q23-26,-15q11,+16p13-11,-16q12-13
-16q13-21,-16q21,-16q21-22,-16q22,-16q22-24
+17q12,+17q21,-17p13,-17p13-11,+19q13
-19q12-13,-19q13,-20p11,-21q21,-21q22,+22q12
+22q12,+22q13,-22q11,-22q13
Common precursor
+1q21-tel,-1p11-q21,+2q31-37,+3p21-14
+3p14-q26,+3q26-27,-3p21,+7p22-q11,+8q21
-8p11-q11,-16p11-q11
+1p21,+2q12,+2q13-14,+2q23-24,+2q24-37,++2q24
++2q31,++2q32,++2q32-35,-5q13,-9p12,-16p12
-Xq28-tel
Common precursor 3
Common precursor 2
+1p34,-1q21,+2q12,+2q13,+2q14,+2q24-31,+2q32
+2q32,+2q33,+2q33-34,+2q34-35,-2q11-12,-2q13
-2q14,+6p22,+6q24-25,-7p11,+10q26,+11q13
-11p11-q11,+12q22-23,+16p11-q11,+17q25
+17q25,+Xq11-12
+1p32,+2q23-24,+2q24-31,+2q32-35,-4p11,+10q22
+14q24,+16q24,-16p13,-16p12,-16p11,-Xq13
+1p21,+1q21-tel,-2q13,+3p21-q26,+3q26-27
-3p21,+7p22-q11,+8p23,+8q21,-10q11,+13q31-34
-15q25,-16p11-q11,+Xq12
+1q24-25,+1q25-31,-1q21,-2q24,+3p14,+3p14-q21
+3q21-22,+3q24-25,+3q25-26,+3q26-27
+7p22-q11,+7q11,+7q11-21,+7q22-31,+7q35
-8p11-q11,+12p13-12,-16p13,-16p11-q11,+17q12
+Xp11-q13
24-6a
24-6b
24-8
24-5

P28

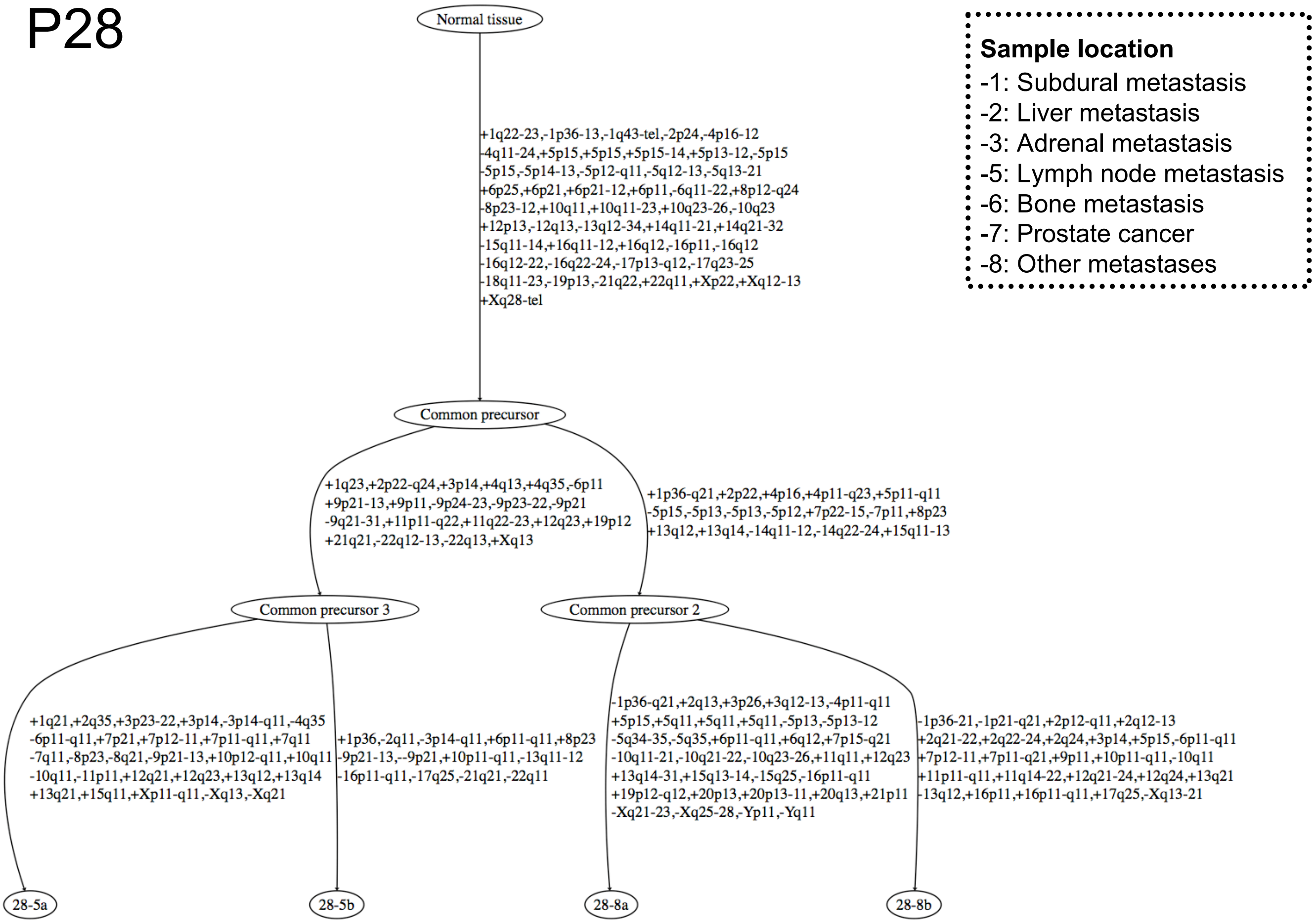
Normal tissue
+1q22-23,-1p36-13,-1q43-tel,-2p24,-4p16-12
-4q11-24,+5p15,+5p15,+5p15-14,+5p13-12,-5p15
-5p15,-5p14-13,-5p12-q11,-5q12-13,-5q13-21
+6p25,+6p21,+6p21-12,+6p11,-6q11-22,+8p12-q24
-8p23-12,+10q11,+10q11-23,+10q23-26,-10q23
+12p13,-12q13,-13q12-34,+14q11-21,+14q21-32
-15q11-14,+16q11-12,+16q12,-16p11,-16q12
-16q12-22,-16q22-24,-17p13-q12,-17q23-25
-18q11-23,-19p13,-21q22,+22q11,+Xp22,+Xq12-13
+Xq28-tel
Common precursor
+1q23,+2p22-q24,+3p14,+4q13,+4q35,-6p11
+9p21-13,+9p11,-9p24-23,-9p23-22,-9p21
-9q21-31,+11p11-q22,+11q22-23,+12q23,+19p12
+21q21,-22q12-13,-22q13,+Xq13
+1p36-q21,+2p22,+4p16,+4p11-q23,+5p11-q11
-5p15,-5p13,-5p13,-5p12,+7p22-15,-7p11,+8p23
+13q12,+13q14,-14q11-12,-14q22-24,+15q11-13
Common precursor 3
Common precursor 2
+1q21,+2q35,+3p23-22,+3p14,-3p14-q11,-4q35
-6p11-q11,+7p21,+7p12-11,+7p11-q11,+7q11
-7q11,-8p23,-8q21,-9p21-13,+10p12-q11,+10q11
-10q11,-11p11,+12q21,+12q23,+13q12,+13q14
+13q21,+15q11,+Xp11-q11,-Xq13,-Xq21
+1p36,-2q11,-3p14-q11,+6p11-q11,+8p23
-9p21-13,--9p21,+10p11-q11,-13q11-12
-16p11-q11,-17q25,-21q21,-22q11
-1p36-q21,+2q13,+3p26,+3q12-13,-4p11-q11
+5p15,+5q11,+5q11,+5q11,-5p13,-5p13-12
-5q34-35,-5q35,+6p11-q11,+6q12,+7p15-q21
-10q11-21,-10q21-22,-10q23-26,+11q11,+12q23
+13q14-31,+15q13-14,-15q25,-16p11-q11
+19p12-q12,+20p13,+20p13-11,+20q13,+21p11
-Xq21-23,-Xq25-28,-Yp11,-Yq11
-1p36-21,-1p21-q21,+2p12-q11,+2q12-13
+2q21-22,+2q22-24,+2q24,+3p14,+5p15,-6p11-q11
+7p12-11,+7p11-q21,+9p11,+10p11-q11,-10q11
+11p11-q11,+11q14-22,+12q21-24,+12q24,+13q21
-13q12,+16p11,+16p11-q11,+17q25,-Xq13-21
28-5a
28-5b
28-8a
28-8b
Sample location
-1: Subdural metastasis
-2: Liver metastasis
-3: Adrenal metastasis
-5: Lymph node metastasis
-6: Bone metastasis
-7: Prostate cancer
-8: Other metastases

P30

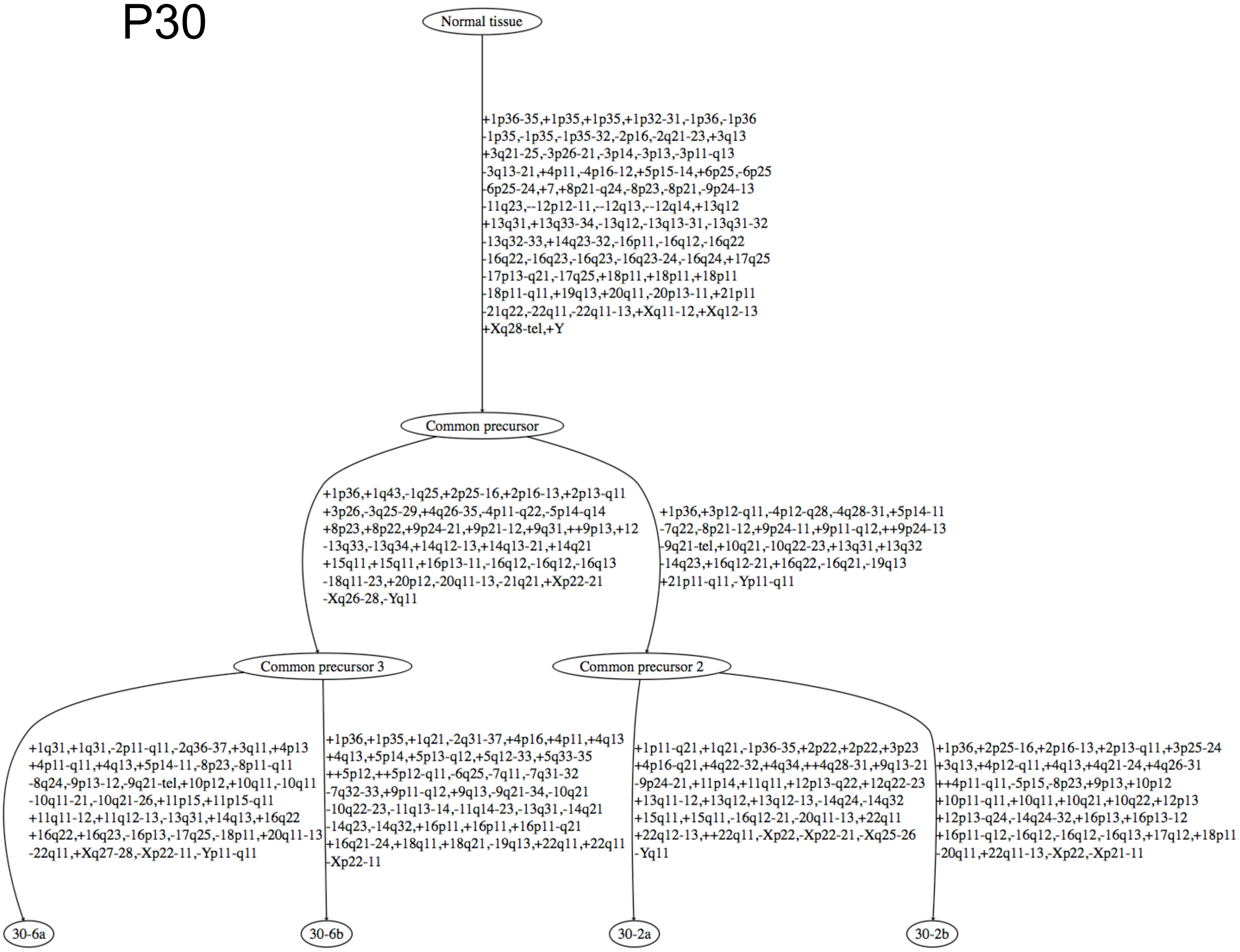

P32

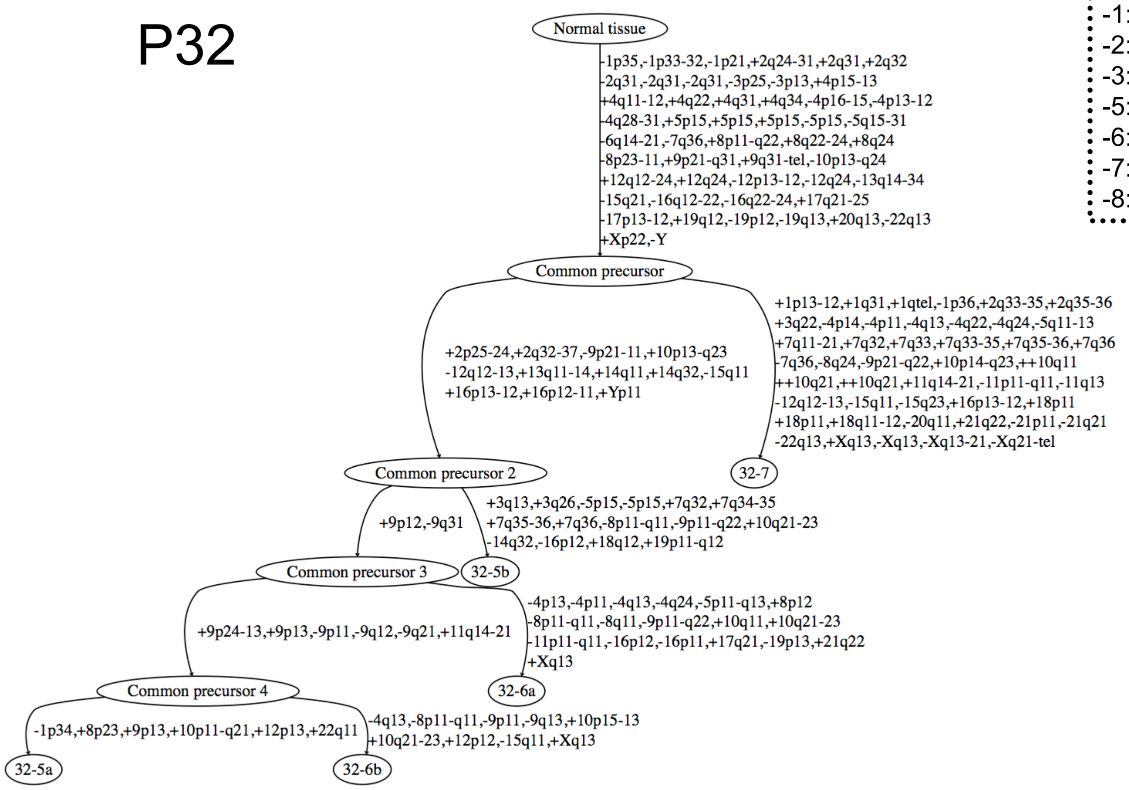

Sample location
- -1: Subdural metastasis
- -2: Liver metastasis
- -3: Adrenal metastasis
- -5: Lymph node metastasis
- -6: Bone metastasis
- -7: Prostate cancer
- -8: Other metastases

P33

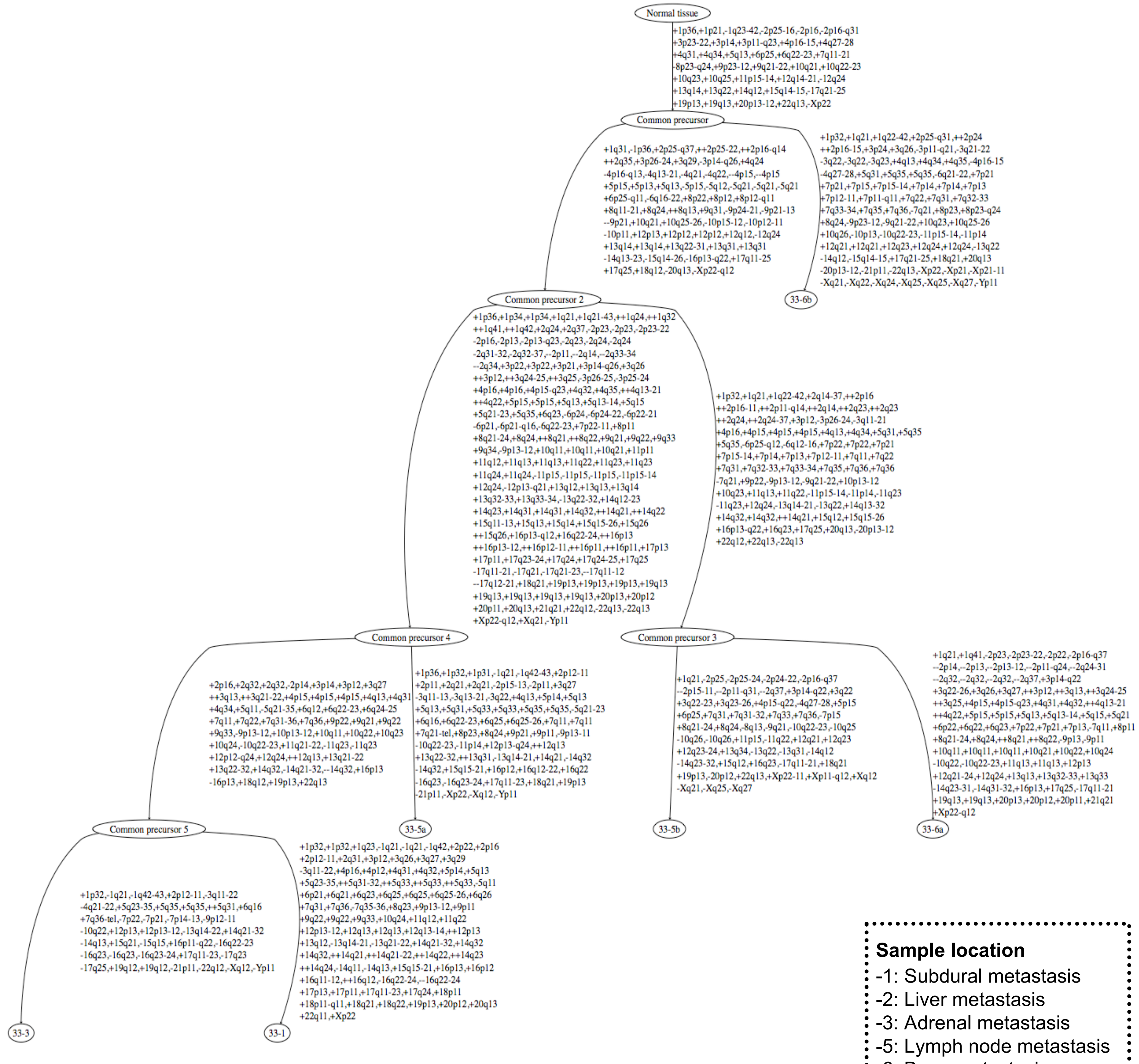

Sample location
- -1: Subdural metastasis
- -2: Liver metastasis
- -3: Adrenal metastasis
- -5: Lymph node metastasis
- -6: Bone metastasis
- -7: Prostate cancer
- -8: Other metastases
