# Supplementary material for: Analysis of the copy number profiles of several tumor samples from the same patient reveals the successive steps in tumorigenesis
Source: Genome Biol. 2010 Jul 22;11(7):R76. doi: 10.1186/gb-2010-11-7-r76 (PMC2926787; doi:10.1186/gb-2010-11-7-r76)
Supplement: Additional file 3 — Clinical data for the 15 bladder samples constituting the reference data set for bladder SNP data. [file gb-2010-11-7-r76-S3.PDF]

Additional data file 3

Clinical data for the 15 bladder samples constituting the reference data set for bladder SNP data

Sample	Sex	Stage	Grade
REF1	M	T3	G3
REF2	M	T3	G3
REF3	M	T2	G3
REF4	M	T1	G3
REF5	M	T1	G3
REF6	M	Ta	G2
REF7	M	T1	G3
REF8	M	T1	G1
REF9	M	T3	G3
REF10	M	T3	G3
REF11	M	T1	G3
REF12	F	T4	G3
REF13	M	T3	G3
REF14	M	T4	G3
REF15	M	T4	G3
