# Supplementary material for: Analysis of the copy number profiles of several tumor samples from the same patient reveals the successive steps in tumorigenesis
Source: Genome Biol. 2010 Jul 22;11(7):R76. doi: 10.1186/gb-2010-11-7-r76 (PMC2926787; doi:10.1186/gb-2010-11-7-r76)
Supplement: Additional file 4 — Segmentation of the profiles and generation of the amplitude vectors. Generation of the amplitude matrix. (a) Discretized copy number profiles of three tumors for a given chromosome (yellow, 'normal copy number'; green, 'loss'; red, 'gain'). The four breakpoints identified in the samples (dashed lines) divide the chromosome into five 'homogeneous segments'. (b) The profiles are equally well represented in a segment matrix, in which the copy number for each segment and each sample is encoded by an integer (-1, loss; 0, normal; 1, gain), in a breakpoint matrix, in which each value represents the difference in copy number between two adjacent segments, or an amplitude matrix, in which 'up' and 'down' breakpoints are distinguished by their position in the vector. A common breakpoint (gray regions) appears as a number of the same sign at the same position in the breakpoint matrix, or as a non-zero number at the same position in the amplitude matrix. [file gb-2010-11-7-r76-S4.PPT]

## Slide 1
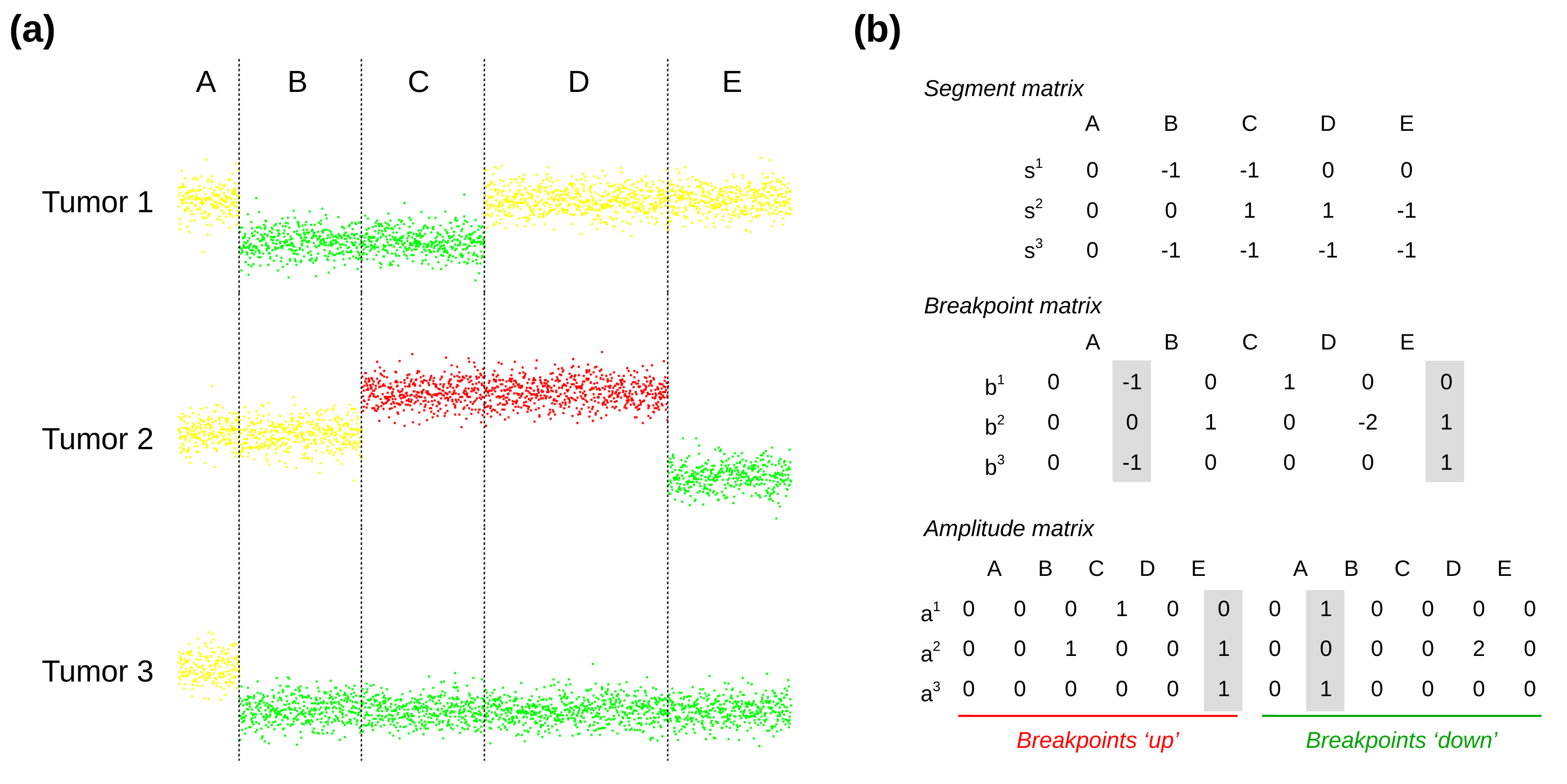

(a)
(b)
A
B
C
D
E
Segment matrix
Tumor 1
Breakpoint matrix
Tumor 2
Amplitude matrix
Tumor 3
Breakpoints ‘up’
Breakpoints ‘down’
