# Supplementary material for: Analysis of the copy number profiles of several tumor samples from the same patient reveals the successive steps in tumorigenesis
Source: Genome Biol. 2010 Jul 22;11(7):R76. doi: 10.1186/gb-2010-11-7-r76 (PMC2926787; doi:10.1186/gb-2010-11-7-r76)
Supplement: Additional file 5 — Two scenarios lead to an unbalanced chromosome in the common precursor. (a) Left: the two tumors independently acquire two different aberrations with a breakpoint in common. Right: the 'up' breakpoint between segments B and C in the common precursor is lost in tumor 2 due to the loss of the neighboring segment C. (b) In both cases, only one breakpoint remains common to both samples, resulting in an unbalanced chromosome for their common precursor. [file gb-2010-11-7-r76-S5.PDF]

(a)

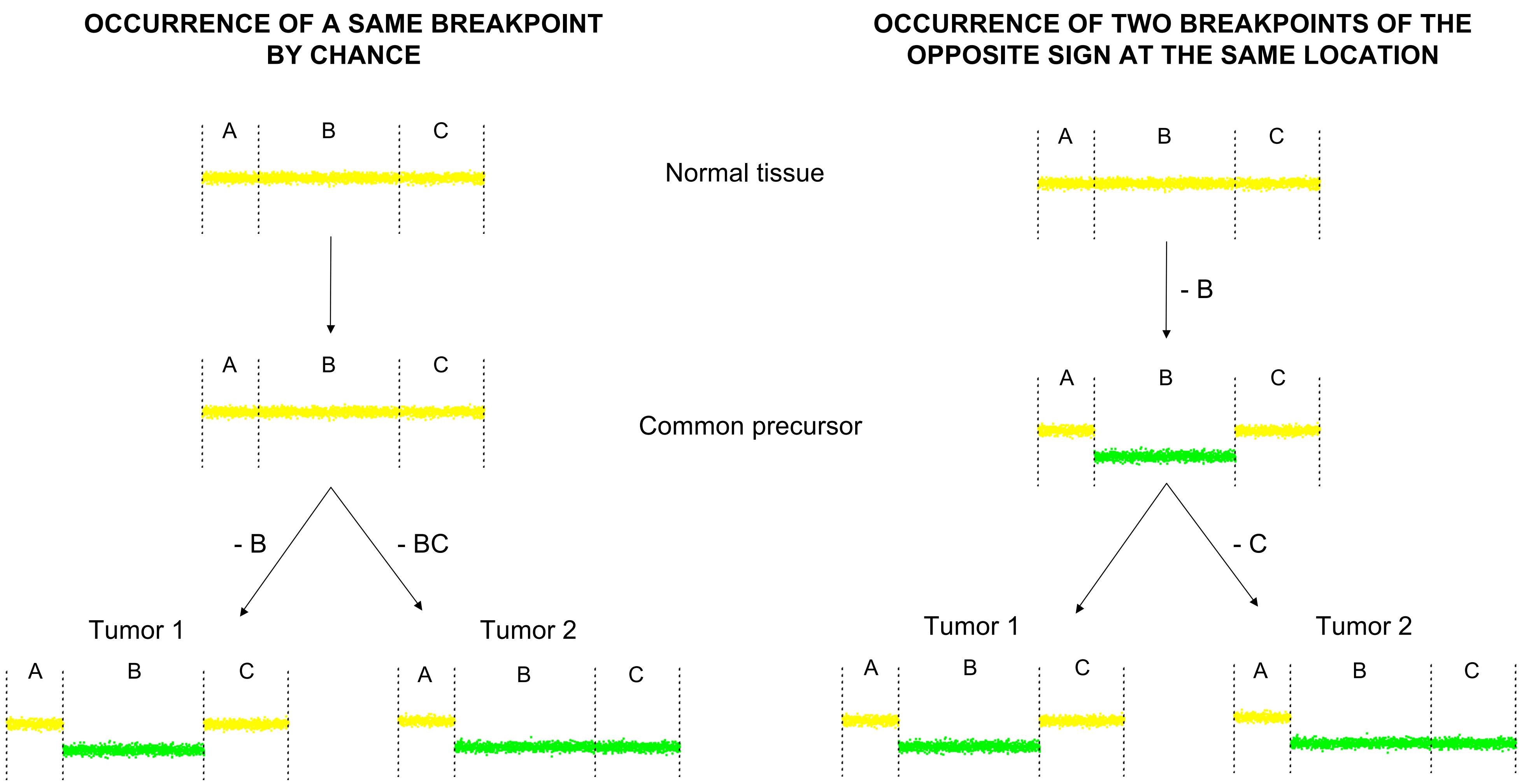

(b)

A B C

b^1	0	-1	1	0
b^2	0	-1	0	1
b^{12}	0	-1	0	0

$$\sum_{k=1}^{4} b_k^{12} = -1$$
