# Supplementary material for: Analysis of the copy number profiles of several tumor samples from the same patient reveals the successive steps in tumorigenesis
Source: Genome Biol. 2010 Jul 22;11(7):R76. doi: 10.1186/gb-2010-11-7-r76 (PMC2926787; doi:10.1186/gb-2010-11-7-r76)
Supplement: Additional file 6 — Correction of an unbalanced chromosome. Detailed procedure of the correction of chromosomes with unbalanced 'up' and 'down' breakpoints. [file gb-2010-11-7-r76-S6.PDF]

Additional data file 6 - Correction of an unbalanced chromosome

This step occurs when the common breakpoints of two nodes do not define a viable copy number profile for a chromosome, due to an imbalance between 'up' and 'down' breakpoints: $\delta = \sum_k b_k^{ij} \neq 0$.

Let us first consider the case in which $|\delta| = 1$. To correct the imbalance, TuMult will either remove a breakpoint of $sign(\delta)$ from the *common breakpoint vector*, or add a breakpoint of $sign(-\delta)$ chosen from the breakpoints specific to one of the two samples. TuMult will choose the breakpoint to be modified on the basis of two criteria:

(i) First, modifying the wrong breakpoint in the common precursor may lead to an impossible scenario. A scenario is considered 'impossible' if a segment deviates from the set of authorized values (-2, -1, 0, 1, 2) in the common precursor, or if a segment with a homozygous deletion (-2) in the common precursor moves to another copy number in a descendant node. These scenarios are immediately discarded.

(ii) Secondly, the 'possible' scenario that minimizes breakpoint usage is selected. Breakpoint usage is defined as the total number of breakpoints affecting the chromosome in the scenario. Let us call a^{ij*} the amplitude vector of the common precursor after correction for breakpoints imbalance. Breakpoint usage is quantified by summing the distance between this common precursor and each node, and the number of breakpoints remaining in the nodes at the *Front* in the next step of the algorithm: $D\left(a^{ij*}, a^i\right) + D\left(a^{ij*}, a^j\right) + \sum_k \max_{f \in Front}\left(a_k^f\right)\left(1 - F_k\right)$. Minimizing this distance penalizes scenarios in which very rare breakpoints occur several times.

The 'possible' scenario minimizing breakpoint usage is therefore selected, and the profile of the common precursor is set to: $CP\left(a^i, a^j\right) = a^{ij*}$

If $|\delta| = n > 1$, the same approach is followed to select the set of *n* breakpoints for which modification leads to a possible scenario minimizing the total number of changes.

(a)

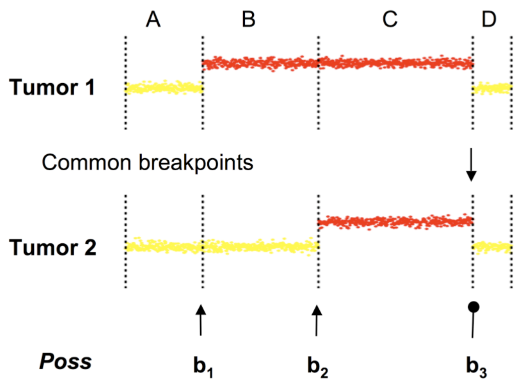

(b)

	A	B	C	D		A	B	C	D	
a^1	0	1	0	0	0	0	0	0	1	0
a^2	0	0	1	0	0	0	0	0	1	0
a^3	0	1	0	0	0	0	0	0	0	1
a^{12}	0	0	0	0	0	0	0	0	1	0
F_k	0.24	0.13	0.09	0.11	0.01	0.03	0.12	0.07	0.04	0.27
k	1	2	3	4	5	6	7	8	9	10

(c)

b1)

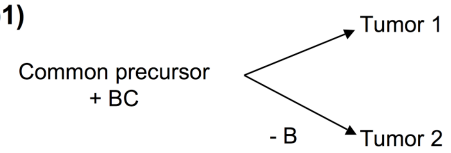

	A	B	C	D		A	B	C	D	
a^{12*}	0	1	0	0	0	0	0	0	1	0
a^3	0	1	0	0	0	0	0	0	0	1
F_k	0.24	0.13	0.09	0.11	0.01	0.03	0.12	0.07	0.04	0.27
k	1	2	3	4	5	6	7	8	9	10

$$D\left(a^{12*},a^1\right)=0$$

$$D\left(a^{12*},a^2\right)=\left(1-F_7\right)+\left(1-F_3\right)=1.79$$

$$\sum_k \max_{f\in Front}\left(a_k^f\right)\left(1-F_k\right)=\sum_k \max\left(a_k^{12*},a_k^3\right)\left(1-F_k\right)=\left(1-F_2\right)+\left(1-F_9\right)+\left(1-F_{10}\right)=2.56$$

$$D\left(a^{12*},a^1\right)+D\left(a^{12*},a^2\right)+\sum_k \max_{f\in Front}\left(a_k^f\right)\left(1-F_k\right)=\mathbf{4.35}$$

b2)

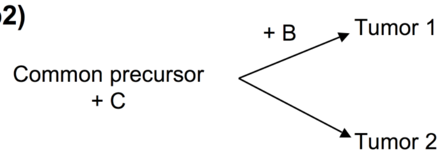

	A	B	C	D		A	B	C	D	
a^{12*}	0	0	1	0	0	0	0	0	1	0
a^3	0	1	0	0	0	0	0	0	0	1
F_k	0.24	0.13	0.09	0.11	0.01	0.03	0.12	0.07	0.04	0.27
k	1	2	3	4	5	6	7	8	9	10

$$D\left(a^{12*},a^1\right)=\left(1-F_2\right)+\left(1-F_8\right)=1.80$$

$$D\left(a^{12*},a^2\right)=0$$

$$\sum_k \max_{f\in Front}\left(a_k^f\right)\left(1-F_k\right)=\sum_k \max\left(a_k^{12*},a_k^3\right)\left(1-F_k\right)=\left(1-F_2\right)+\left(1-F_3\right)+\left(1-F_9\right)+\left(1-F_{10}\right)=3.47$$

$$D\left(a^{12*},a^1\right)+D\left(a^{12*},a^2\right)+\sum_k \max_{f\in Front}\left(a_k^f\right)\left(1-F_k\right)=\mathbf{5.27}$$

b3)

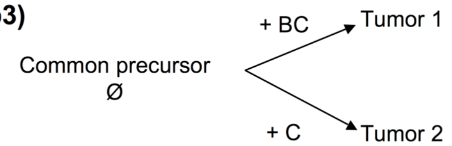

	A	B	C	D		A	B	C	D	
a^{12*}	0	0	0	0	0	0	0	0	0	0
a^3	0	1	0	0	0	0	0	0	0	1
F_k	0.24	0.13	0.09	0.11	0.01	0.03	0.12	0.07	0.04	0.27
k	1	2	3	4	5	6	7	8	9	10

$$D\left(a^{12*},a^1\right)=\left(1-F_2\right)+\left(1-F_9\right)=1.83$$

$$D\left(a^{12*},a^2\right)=\left(1-F_3\right)+\left(1-F_9\right)=1.87$$

$$\sum_k \max_{f\in Front}\left(a_k^f\right)\left(1-F_k\right)=\sum_k \max\left(a_k^{12*},a_k^3\right)\left(1-F_k\right)=\left(1-F_2\right)+\left(1-F_{10}\right)=1.60$$

$$D\left(a^{12*},a^1\right)+D\left(a^{12*},a^2\right)+\sum_k \max_{f\in Front}\left(a_k^f\right)\left(1-F_k\right)=\left(1-F_2\right)+\left(1-F_{10}\right)=\mathbf{5.30}$$

Figure 1 – Correction of an unbalanced chromosome

This figure shows an example of correction for an unbalanced chromosome. In this example, tumors 1 and 2 are joined in the tree and the profile of their common precursor is being reconstructed. A third tumor (tumor 3) remains in the *Front* at this step of tree reconstruction.

(a) Tumors 1 and 2 have only one breakpoint in common, leading to an unbalanced chromosome in the common precursor: $\delta = \sum_k b_k^{12} = -1$. There are three possible corrections (*Poss*) for this chromosome: add an 'up' breakpoint from among the breakpoints present in only one of the tumors (b_1 or b_2), or remove the common breakpoint from the common precursor (b_3).

(b) Amplitude vectors of the three tumors, and the *common amplitude vector* of tumors 1 and 2, a^{12}. The frequency F_k of each breakpoint in the reference data set is also indicated.

(c) Scenarios corresponding to each choice. For each case, the subtree with the common precursor and its two descendant nodes is represented, together with the corrected *common amplitude vector*, and the details of the calculation of breakpoint usage. Adding b_1 or b_2 leads to a scenario in which a breakpoint of the common precursor is erased by a subsequent aberration targeting the neighboring segment. Removing b_3 means the two gains occured independently in the two samples and share a common breakpoint by chance. None of these three scenarios displays an impossible feature (such as a segment deviating from authorized values), hence breakpoint usage is calculated for all three scenarios. This is achieved by adding the distances between the common precursor and each descendant node to the number of breakpoints remaining in the *Front* for the next step. Here, the two nodes that will remain in the *Front* are the common precursor and tumor 3.

Note that in scenario b_1, the common precursor and tumor 3 share a common breakpoint. This breakpoint is therefore counted only once, in $\sum_k \max_{f \in Front}\left(a_k^f\right)\left(1 - F_k\right)$, leading to a smaller breakpoint usage for this scenario (only 5 breakpoints in total,

versus 6 for b_2 and b_3). Thus, if a breakpoint is present in only one of the two nodes being joined, but is also present in another node in the *Front*, it will generally be included in the common precursor. Inded, such a breakpoint is more likely to have occurred early in the tree and to have been removed by a subsequent event in one of the two nodes being joined.

In this example, breakpoint b_1 is added to the profile of the common precursor.
